# Bisindole Alkaloids from the *Alstonia* Species: Recent Isolation, Bioactivity, Biosynthesis, and Synthesis †

**DOI:** 10.3390/molecules26113459

**Published:** 2021-06-07

**Authors:** Kamal P. Pandey, Md Toufiqur Rahman, James M. Cook

**Affiliations:** 1 Department of Chemistry and Biochemistry, University of Wisconsin Milwaukee, Milwaukee, WI 53211, USA; kppandey@uwm.edu; 2 RTI International, Center for Drug Discovery, Research Triangle Park, Durham, NC 27709, USA; mrahman@rti.org

**Keywords:** bisindole synthesis, biosynthesis, bioactivity, *Alstonia*, Apocynaceae, sarpagine, macroline, ajmaline, pleiocarpamine, partial, biomimetic or total synthesis

## Abstract

Bisindoles are structurally complex dimers and are intriguing targets for partial and total synthesis. They exhibit stronger biological activity than their corresponding monomeric units. Alkaloids, including those containing C-19 methyl-substitution in their monomeric units, their synthetic derivatives, and their mismatched pairs can be attractive targets for synthesis and may unlock better drug targets. We herein discuss the isolation of bisindoles from various *Alstonia* species, their bioactivity, putative biosynthesis, and synthesis. The total synthesis of macralstonidine, macralstonine, *O*-acetylmacralstonine, and dispegatrine, as well as the partial synthesis of alstonisidine, villalstonine, and macrocarpamine are also discussed in this review. The completion of the total synthesis of pleiocarpamine by Sato et al. completes the formal synthesis of the latter two bisindoles.

## 1. Introduction

Nature has been a substantial and sustainable pool of biologically active compounds. Since ancient times natural product extracts (in crude form) have been used in traditional and folk medicines in many countries. In modern times pure (isolated) natural products and their derivatives play an important role in drug discovery, as indicated by their prevalence in approved drugs for clinical use. Out of the 1881 newly FDA-approved drugs over the last four decades (1 January 1981 to 30 September 2019), a significant portion comprising 506 (26.9%) were either natural products or derived from or inspired by natural products [1]. It is expected that the advent of modern and innovative technologies such as computational software, cheminformatics, artificial intelligence, automation, and quantum computing will further boost natural product-based drug discovery. A synergy among these technological milestones would accelerate *hit to lead to clinic* pathways of drug discovery, and natural products are expected to remain an important source [2]. Moreover, pharmacophores and their unique stereochemical interactions with natural products may stimulate more demanding targets such as protein–protein interactions in the near future and open up a new avenue in modern drug discovery [3]. The majority of biologically active natural products are produced in plants, known traditionally as medicinal plants.

*Alstonia*, a major genus in the Apocynaceae family of plants, has nearly 155 species and is found all over the world [4]. Robert Brown named it in 1811 in honor of Charles Alston (1685–1760), an eminent botanist at the University of Edinburgh [4]. The *Alstonia* genus’ trees and shrubs are prevalent in the tropical and subtropical parts of Africa, Asia, and Australia [5]. They contribute significant pharmacological activity, including anticancer, antileishmanial, antimalarial, antitussive, antiviral, antiarthritic, and antibacterial activities [4,5,6,7,8,9]. Several parts of the plants including bark, twigs, and leaves have been traditionally used to treat diseases. Their useful biological activity has aroused profound interest, as indicated by the increasing number of publications and patents each year. To date, more than 300 patents have been filed under the *Alstonia* genus with various formulations [4]. Notably, 157 different monoterpenoid indole alkaloids have been isolated from this genus between 2010 to 2020 [10].

Alkaloids, the most important class of natural products with structural diversity and significant pharmacological effects, are mainly found in higher plants such as the Apocynaceae, Ranunculaceae, Papaveraceae, and Leguminosae families [11]. These natural products, along with flavonoids, fatty acids, etc., are the major classes of secondary metabolites that are believed to be parts of the plants’ defense mechanism. To date, many monoterpenoid indole and bisindole alkaloids have been found in the *Alstonia* genus [4]. Modern clinical application of many of these alkaloids are similar to their traditional or folklore applications; for example, cocaine and morphine were used as anesthetics while caffeine and nicotine were used as stimulants [12]. Recently, Fielding et al. illustrated that several anti-coronavirus alkaloids showed potential therapeutic value against severe acute respiratory syndrome coronavirus-2 (SARS-CoV-2) in their in silico studies [9].

The indole motif is present in many naturally occurring and biologically active compounds. Some of them have been used in the clinic. In addition, there are numerous examples of synthetic compounds with useful medicinal properties that bear the indole moiety. Consequently, the indole scaffold is one of the few “privileged structures” in modern medicinal chemistry and drug discovery [13,14]. The prevalence of bioactivity of indole-containing molecules may be attributed to their similarity to the essential amino acid tryptophan, as well as important biomolecules such as tryptamine and serotonin. Many plant extracts, which likely include alkaloids, have been used from time immemorial in folk medicines for fever, general weakness, dysentery, pain, liver diseases, gastrointestinal diseases, and cancer [15]. Currently, there are many indole alkaloid-based marketed drugs such as sumatriptan for the treatment of migraine; vincristine and vinblastine for the treatment of various cancers, including leukemia and lung cancer; as well as reserpine for the treatment of hypertension and to decrease severe agitation in patients with mental disorders [16].

Bisindole alkaloids are naturally occurring alkaloids containing two indole nuclei. These bisindoles are the products of late-stage biosynthetic processes in higher plants by combining two monomeric units. Depending on the monomeric units involved, bisindoles can be a homo- or heterodimer. As a result, bisindole alkaloids comprise much higher structural complexity than both of the monomeric units that comprise them. As mentioned, bioactive bisindoles are usually more potent than the monomeric units that comprise them, although the mechanism of action is not known [6]. More structural diversity can be achieved via bisindole-based drug discovery by changing both monomeric units to furnish unnatural and *pseudo*-natural alkaloids. As such, bisindole alkaloids offer a large pool of natural, semi-natural, and chimeric drug candidates that have greater drug-like characteristics. As a result, due to their important biological applications and complex structural features, bisindole alkaloids have engendered the profound interest of synthetic organic and medicinal chemists, computational chemists, and chemical biologists. Compared to the previously published review [6] from this group, the most recent and updated reports on the isolation, important bioactivity, biosynthetic studies, and synthesis of bisindole alkaloids isolated from *Alstonia* containing either sarpagine, macroline, ajmaline, or pleiocarpamine units form the basis of this review. Moreover, the recent total synthesis of *rac*-pleiocarpamine (a monomeric unit present in several bisindoles) by Sato et al. [17], which resulted in the formal total synthesis of villalstonine and macrocarpamine, the bioactive C-19 methyl-substituted sarpagine/macroline/ajmaline indole alkaloids, their unnatural enantiomers, and mismatched pairs to form potential bisindoles alkaloids, is also discussed in this review.

## 2. Occurrence and Isolation of Bisindoles from *Alstonia* Species

A list of representative families to which the monomeric indole-based units belong, which comprise bisindole alkaloids from *Alstonia,* is shown in Table 1. In some cases, a monomeric unit itself occurs as an alkaloid, i.e., has been isolated separately and is known by its trivial or given name. Bisindole alkaloids that contain the illustrated monomeric bases are listed in Table 2. Among various species of the *Alstonia* genus, *A. macrophylla* and *A. angustifolia* are the two major sources of bisindole alkaloids discussed herein (vide infra, Table 3). (+)-Alstomacroline **1**, a bisindole alkaloid consisting of a macroline and an ajmaline unit, was isolated from the bark *of A. macrophylla* [18] and the leaves, stem-bark, and root-bark extracts of *A. scholaris, A. glaucescens, and A. macrophylla* [19,20]. (+)-Alstomacrophylline **2** (macroline–macroline-type) was isolated from the bark *of A. macrophylla* [18] and the leaves, stem-bark, and root-bark extracts of *A. scholaris, A. glaucescens, and A. macrophylla* [20]. (-) Alstonisidine **3**, which contains a quebrachidine **4** and a macroline **5** unit, was isolated from the bark of *A. muelleriana* [21,22]. The structure of (-)-alstonisidine **3** was confirmed by X-ray crystallographic data [23]. Yeap et al. recently isolated seven novel bisindoles from the methanol extract of the stem-bark of Malayan *A. penangiana* [5]. This includes (-)-angustilongine E **6**, (-)-angustilongine F **7**, (+)-angustilongine G **8**, (+)-angustilongine H **9**, (+)-angustilongine J **10**, (+)-angustilongine K **11**, and (-)-angustilongine L **12** (macroline–pleiocarpamine type). (+)-Angustilongine K **11** was converted into (+)-di-*O*-acetylangustilongine K **13** by stirring it with 10 equivalents of pyridine and 15 equivalents of acetic anhydride for 6 h at room temperature in **95%** yield [5]. Among those, angustilongine G **8** and angustilongine H **9** are C-19 methyl substituted [24] bisindoles. The structures of the angustilongines were confirmed by various spectroscopic data, including ^1^H NMR, ^13^C NMR, 2D NMR, IR, and HRMS by Yeap et al. [5]. Angustilongine E **6**, angustilongine F **7**, angustilongine G **8**, angustilongine H **9**, angustilongine J **10**, and angustilongine K **11** are macroline–sarpagine coupled bisindoles. Angustilongine G **8** and angustilongine H **9** differ in stereochemistry only at the C-20 position.

Two macroline units are contained in (-)-lumusidine A **14**, (-)-lumusidine B **15**, (-)-lumusidine C **16**, and (-)-lumusidine D **17** bisindoles. They were isolated from the stem-bark of *A. macrophylla* and the structures were confirmed via NMR spectroscopy, mass spectrometry, UV spectroscopy, and X-ray crystallography [18]. After isolation, the group of Kam et al. converted oily (-)-lumusidine A **14**, (-)-lumusidine B **15**, and (-)-lumusidine D **17** into the corresponding crystalline dimethyl diiodide salts (structures not shown) by treatment with an excess of iodomethane for 24 h. The crystalline salts were employed to obtain X-ray crystallographic data to elucidate the exact stereochemical confirmation [18]. (-)-Lumusidine D **17** is also known as thungfaine [25]. (+)-Lumutinine A **18**, (-)-lumutinine B **19**, (+)-lumutinine C **20**, and (+)-lumutinine D **21** are linearly fused bisindoles isolated from the stem-bark of *A. macrophylla* as a light yellowish oil [26]. (+)-Lumutinine A **18** and (-)-lumutinine B **19** are macroline–macroline-type bisindoles, while (+)-lumutinine C **20**, (+)-lumutinine D **21**, and (+)-lumutinine E **22** are macroline–sarpagine-type bisindoles. The structures of the lumutinines were elucidated using spectroscopic means including 1D and 2D NMR, IR, as well as mass spectrometric analysis [26]. The structure of (+)-lumutinine D **21** was confirmed by X-ray crystallographic data [27]. (+)-Lumutinine E **22**, a macroline–sarpagine-type bisindole, was isolated from the stem-bark of *A. angustifolia* [28]. 

(+)-Macralstonidine **23** (macroline–sarpagine-type) was isolated from the bark of *A. macrophylla* [29,30], as well as from *A. somersentenis* [29] and *A. spectabilis* [31]. (+)-Macralstonine **24** was isolated from the leaves, stem-bark, and root-bark extracts of *A. scholaris, A. glaucescens, and A. macrophylla* extracts [20], *A. macrophylla* [29,30,32,33,34], *A. muelleriana* [35], *A. angustifolia* [36], as well as from *A. glabriflora* [31]. The structure of (+)-macralstonine **24** was confirmed by various NMR spectroscopy, mass spectrometry, and X-ray crystallography [33]. The (+)-macralstonine **24**-related bisindole, (+)-*O*-acetylmacralstonine **25,** was isolated from the leaves, stem-bark, and root-bark extracts of *A. scholaris, A. glaucescens, and A. macrophylla* [20]. Also, (+)-*O*-methylmacralstonine **26** was isolated from the leaves, stem-bark, and root-bark of *A. scholaris, A. glaucescens, and A. macrophylla* extracts [20]. (-)-Anhydromacralstonine **27** was isolated from the stem-bark of *A. angustiloba* [28] and contains (-)-alstophylline **28** and (+)-macroline **5** as monomeric units. Another (-)-alstophylline **28** and (+)-macroline **5** monomeric bisindole, (+)-*Des-N’a*-methylanhydromacralstonine **30,** was isolated from the bark of *A. muelleriana* [20,35], the stem-bark of *A. angustifolia* [33], and *A. glabriflora* [31]. (+)-Macrocarpamine **31**, a hetero-dimeric bisindole containing a (+)-pleiocarpamine **32** and (-)-anhydromacrosalhinemethine **33** monomeric unit was isolated from the leaves, stem-bark, and root-bark of *A. scholaris, A. glaucescens, and A. macrophylla* [20], as well as the stem-bark of *A. angustifolia* [28]. 10-Methoxymacrocarpamine **34** and 10-methoxymacrocarpamine *N4′*-oxide **35** are structurally related bisindoles. These bisindoles were isolated from *A. angustifolia* leaves [36].

Lim et al. [33] reported the isolation of new macroline–macroline-type bisindoles, (-)-perhentidine A **36**, (-)-perhentidine B **37**, and (-)-perhentidine C **38** from the ethanolic extract of the stem-bark of Malayan *A. macrophylla* and *A. angustifolia.* The structurally related bisindole, (-)-perhentinine **39** (macroline–macroline-type), was isolated from the stem-bark and leaves of *A. macrophylla* and the leaves of *A. angustifolia* [18]. The exact structure of (-)-perhentinine **39** was confirmed by X-ray crystallography, converting it into the dimethyl diiodide salt by treating it with an excess of iodomethane. The X-ray crystallographic data of (-)-perhentinine **39** also helped in the structural characterizations of perhentidines A–C (**36–38**) [33]. Tan et al. isolated three macroline–sarpagine-type bisindoles: (+)-perhentisine A **40**, (-)-perhentisine B **41**, and (+)-perhentisine C **42** from the stem-bark of *A. angustifolia* as a light yellow-colored oil together with other bisindoles [28]. The structures of these bisindoles were also elucidated using various NMR and MS techniques [28]. (+)-Villastonine **43**, a macroline–pleiocarpamine-type bisindole, was isolated from the stem-bark, root-bark, and leaves of various *Alstonia* species, including *A. spectabilis* [29] and *A. muelleriana* [37] by LeQuesne et al., *A. macrophylla* [30,32,38], and *A. angustifolia* [36,39]. Schmid et al. elucidated the structure of (+)-villalstonine **43** by spectroscopic means, accompanied by degradation, and Nordman et al. confirmed the structure by X-ray crystallography [38,40].

**Table 3:** Isolation and plant morphology of bisindole alkaloids from *Alstonia* species.

Bisindoles	Types of Monomeric Units Present in Bisindoles	*Alstonia* Species	Morphology and References
(+)-Alstomacroline **1**	Macroline-sarpagine	*A. scholaris, A. glaucescens*, and *A. macrophylla* extracts	Leaves, stem-bark, and root-bark [19,20]
		*A. macrophylla*	Bark [18]
(+)-Alstomacrophylline **2**	Macroline-macroline	*A. macrophylla*	Bark [19]
		*A. scholaris*, *A. glaucescens*, and *A. macrophylla* extracts	Leaves, stem-bark, and root-bark [20]
(−) Alstonisidine **3**	Ajmaline-macroline	*A. muelleriana*	Bark [21,22]
(−)-Angustilongine E **6**	Macroline-sarpagine	*A. penangiana*	Stem-bark [5]
(−)-Angustilongine F **7**	Macroline-sarpagine	*A. penangiana*	Stem-bark [5]
(+)-Angustilongine G **8**	Macroline-sarpagine	*A. penangiana*	Stem-bark [5]
(+)-Angustilongine H **9**	Macroline-sarpagine	*A. penangiana*	Stem-bark [5]
(+)-Angustilongine J **10**	Macroline-sarpagine	*A. penangiana*	Stem-bark [5]
(+)-Angustilongine K **11**	Macroline-sarpagine	*A. penangiana*	Stem-bark [5]
(−)-Angustilongine L **12**	Macroline-pleiocarpamine	*A. penangiana*	Stem-bark [5]
(−)-Anhydromacralstonine **27**	Macroline-macroline	*A. angustifolia*	Stem-bark [28]
(+)-*Des-N’a*-Methylanhydromacralstonine **30**	Macroline-macroline	*A. muelleriana*	Bark [20]
		*A. angustifolia*	Stem-bark [33]
		*A. muelleriana*	Leaves, stem-bark and root-bark [20,35]
(−)-Lumusidine A **14**	Macroline-macroline	*A. macrophylla*	Stem-bark [18]
(−)-Lumusidine B **15**	Macroline-macroline	*A. macrophylla*	Stem-bark [18]
(−)-Lumusidine C **16**	Macroline-macroline	*A. macrophylla*	Stem-bark [18]
(−)-Lumusidine D **17**	Macroline-macroline	*A. macrophylla*	Stem-bark [18]
(+)-Lumutinine A **18**	Macroline-macroline	*A. macrophylla*	Stem-bark [26]
(−)-Lumutinine B **19**	Macroline-macroline	*A. macrophylla*	Stem-bark [26]
(+)-Lumutinine C **20**	Macroline-sarpagine	*A. macrophylla*	Stem-bark [26]
(+)-Lumutinine D **19**	Macroline-sarpagine	*A. macrophylla*	Stem-bark [26]
(+)-Lumutinine E **21**	Macroline-sarpagine	*A. angustifolia*	Stem-bark [28]
(+)-Macralstonidine **23**	Macroline-sarpagine	*A. macrophylla*	Bark [29,30]
		*A. somersentenis*	Bark [29]
		*A. spectabilis*	Bark [31]
(+)-Macralstonine **24**	Macroline-macroline	*A. scholaris, A. glaucescens,* and *A. macrophylla* extracts	Leaves, stem-bark and root-bark [19]
		*A. macrophylla*	[29,30,32,33,34]
		*A. angustifolia*	[36]
		*A. muelleriana*	[35]
		*A. glabriflora* Mgf.	[31]
(+)-*O*-Acetylmacralstonine **25**	Macroline-macroline	*A. scholaris, A. glaucescens,* and *A. macrophylla* extracts	[20]
(+)-*O*-Methylmacralstonine **26**	Macroline-macroline	*A. scholaris, A. glaucescens,* and *A. macrophylla* extracts	[20]
(+)-Macrocarpamine **31**	Macroline-pleiocarpamine	*A. scholaris, A. glaucescens*, and *A. macrophylla* extracts	Leaves, stem-bark, and root-bark [20]
		*A. angustifolia*	Stem-bark [28]
10-Methoxy macrocarpamine **34**	Macroline-pleiocarpamine	*A. angustifolia*	Leaves [41]
10-Methoxy macrocarpamine 4′-*N*-oxide **35**	Macroline-pleiocarpamine	*A. angustifolia*	Leaves [41]
(−)-Perhentidine A **36**	Macroline-macroline	*A. macrophylla* and *A. angustifolia*	Stem-bark [28,33]
(−)-Perhentidine B **37**	Macroline-macroline	*A. macrophylla* and *A. angustifolia*	Stem-bark [33]
(−)-Perhentidine C **38**	Macroline-macroline	*A. macrophylla* and *A. angustifolia*	Stem-bark [28,33]
(−)-Perhentinine **39**	Macroline-macroline	*A. macrophylla*	Stem-bark [18]
		*A. angustifolia*	Leaves [18]
		*A. macrophylla*	Leaves [18]
(+)-Perhentisine A **40**	Macroline-sarpagine	*A. angustifolia*	Stem-bark [28]
(−)-Perhentisine B **41**	Macroline-sarpagine	*A. angustifolia*	Stem-bark [28]
(+)-Perhentisine C **42**	Macroline-sarpagine	*A. angustifolia*	Stem-bark [28]
		*A. muelleriana*	Leaves and stem-bark [37]
(+)-Villalstonidine A **47**	Macroline-pleiocarpamine	*A. angustifolia*	Stem-bark [28]
(+)-Villalstonidine B **48**	Macroline-pleiocarpamine	*A. angustifolia*	Stem-bark [28]
		*A. macrophylla*	Stem-bark [27]
(+)-Villalstonidine C **49**	Macroline-pleiocarpamine	*A. angustifolia*	Stem-bark [28]
(+)-Villalstonidine D **50**	Macroline-pleiocarpamine	*A. angustifolia*	Stem-bark [28]
(+)-Villalstonidine E **51**	Macroline-pleiocarpamine	*A. angustifolia*	Stem-bark [28]
(+)-Villalstonidine F **52**	Macroline-pleiocarpamine	*A. macrophylla*	Stem-bark [27]
(+)-Villalstonine **43**	Macroline-pleiocarpamine	*A. angustifolia*	Leaves and stem-bark [28,36,39,41]
		*A. macrophylla*	[41]
		*A. villosa*	[41]
		*A. verticillosa*	[41]
		*A. somersentensis*	[41]
Villalstonine *N(4)*-oxide **44**	Macroline-pleiocarpamine	*A. angustifolia*	Stem-bark [28]
		*A. scholaris, A. glaucescens,* and *A. macrophylla* extracts	Leaves, stem-bark, and root-bark [20,30,32,38]
(+)-10-Methoxy villalstonine **45**	Macroline-pleiocarpamine	*A. angustifolia*	Leaves [41]
10-Methoxy villalstonine 4′-*N*-oxide **46**	Macroline-pleiocarpamine	*A. angustifolia*	Leaves [41]

Villalstonine *N*(4)-oxide **44** was isolated from the stem-bark of *A. angustifolia* [28]. It was also isolated from the leaves, stem-bark, and root-bark of *A. scholaris, A. glaucescens, and A. macrophylla* extracts [20]. Moreover, two villalstonine **44**-related bisindoles, (+)-10-methoxy villalstonine **45** and 10-methoxy villalstonine *N*(4)-oxide **46**, were isolated from the leaves of *A. angustifolia* [41]. Villalstonidine A **47**, villalstonidine B **48**, villalstonidine C **49**, villalstonidine D **50**, villalstonidine E **51**, (+)-villalstonidine F **52**, and villalstonine *N*(4)-oxide **44** are macroline–pleiocarpamine-type bisindoles and are close in structure to villalstonine **43**. Villalstonidines A–D (**47–50)** were isolated from the stem-bark of *A. angustifolia* [28]. Additionally, (+)-villalstonidine F **52** {*N*(1)-demethylderivative of villalstonine **43**} and (+)-villalstonidine B **48** were isolated from the stem-bark of *A. macrophylla* [27]. 

## 3. Bioactivity of Bisindole Alkaloids from *Alstonia* Species

Studies from various groups have shown that bisindoles have anticancer activity in different cell lines, including HT-29 and vincristine-resistant KB/VJ300 cells. Bisindoles are also reported to have other biological activities, including antiprotozoal activity against *Plasmodium falciparum* and antileishmanial activity against promastigotes of *Entamoeba histolytica*. The reported biological activity of bisindoles from various *Alstonia* species including the semi-synthetic derivatives reviewed herein are listed in Table 4. (+)-Alstomacroline **1** and (+)-alstomacrophylline **2** bisindoles were active against the K1 (multi-drug resistant) strain of *P. falciparum* with an IC_50_ 1.12 ± 0.35 μM and IC_50_ 1.10 ± 0.30 μM, respectively [42]. Newly isolated macroline-sarpagine-type bisindoles, (−)-angustilongines E **6**, (-)-angustilongine F **7**, (+)-angustilongine G **8**, (+)-angustilongine H **9**, (+)-angustilongine J **10**, and (+)-angustilongine K **11** showed in vitro growth inhibitory activity against human cancer cell lines, inclusive of KB, vincristine-resistant strains of KB, HCCT 116, PC-3, MDA-MB-231, LNCaP, MCF7, HT-29, and A549 cells with IC_50_ values ranging from 0.02 to 9.0 μM in the study from the group of Kam [5]. Kam et al. [18] also reported the anticancer activity of (−)-lumusidine A **14**, (−)-lumusidine B **15**, (−)-lumusidine C **16**, and (−)-lumusidine D **17**. These bisindoles were cytotoxic against KB/VJ300 cells ranging from IC_50_ 0.16 to 5.03 μg/mL (μM) values with 0.12 μM vincristine added [18]. Lumutinine A **18**, lumutinine B **19**, lumutinine C **20**, lumutinine D **21**, and lumutinine E **22** exhibited moderate anticancer activity against KB/VJ300 cells ranging in IC_50_ value from 0.10 to 4.61 μg/mL (μM) again with 0.12 μM vincristine added in the studies from the same group (Kam) [18]. 

(+)-Macralstonine **24** was active against the K1 strain of *P. falciparum* (IC_50_ 8.92 ± 2.95 μM). Notably, the derivatives of (+)-macralstonine **24** were more active than (+)-macralstonine **24** itself. (+)-*O*-methyl macralstonine **26** and (+)-*O*-acetyl macralstonine **25** demonstrated more potent activity against the K1 strain of *P. falciparum* with IC_50_ values of 0.85 ± 0.20 μM and IC_50_ 0.53 ± 0.09 μM, respectively [20]. Likely, the functionalization facilitated the transportation of these bisindoles through the cell membranes of parasites and red blood cells, which would have enhanced the activity as lipophilicity rose [20]. (+)-*O*-acetyl macralstonine **25** and (+)-alstomacroline **1** were also somewhat active against the T9-96 strain of *P. falciparum* with IC_50_ values of 12.4 and 10.2 μM, respectively [20]. Heterodimeric alkaloid (−)-anhydromacralstonine **27** showed moderate cytotoxicity (IC_50_ value of 0.44 μg/mL (μM) in KB/VJ300 cells with 0.12 μM of vincristine added [18]). Another semisynthetic analog of macralstonine **24**, the related *O*-acetyl-*E-seco*-macralstonine **53**, showed strong anticancer activity. It was prepared by the reaction of macralstonine **24** with acetic anhydride/pyridine in DCM [18]. It demonstrated potent activity against vincristine-resistant KB/VJ300 cells with an IC_50_ value of 0.27 μg/mL (μM), with 0.12 μM of vincristine added to the assay [18]. (+)-Macralstonidine **23** was found to exhibit moderately active anticancer activity. It was active against KB/VJ300 cells with an IC_50_ value of 0.13 μg/mL (μM) [18].

(−)-Macrocarpamine **31** exhibited antiprotozoal and anticancer activity in various studies. It showed significant antiprotozoal activity in vitro in studies from Wright et al. against *E. histolytica* and *P. falciparum* with ED_50_ values of 8.12 (95% C.I.) μM and 9.36 (95% C.I.) μM, respectively [43]. Keawpradub et al. reported significant activity of (−)-macrocarpamine **31** against the K1 strain of *P. falciparum* with an IC_50_ value of 0.36 μM [20]. Furthermore, (−)-macrocarpamine **31** showed antimalarial activity against the T9-96 strain of *P. falciparum* with an IC_50_ > 39 μM. The ancient folklore use of the extracts from *A. angustifolia* in Malaya for treatment of malaria and dysentery is supported by these in vitro studies [44]. (−)-Macrocarpamine **31** was strongly cytotoxic in KB/VJ300 cells with an IC_50_ value of 0.53 μg/mL (μM) [18]. (−)-Perhentidine A **36** and (−)-perhentidine B **37** showed strong cytotoxicity against KB/VJ300 cells with IC_50_ values of 2.29 and 0.84 μg/mL (μM), respectively [18]. *O*-Acetylperhentidine A **54** and *O*-acetylperhentidine B **55** also exhibited strong cytotoxicity against KB/VJ300 cells with IC_50_ values of 0.36 and 0.28 μg/mL (μM), respectively [18]. (−)-Perhentidine A **36** and (−)-perhentidine B **37** were treated individually by dropwise addition of acetic anhydride in a py/DCM solution, followed by stirring at room temperature for 2 h to furnish the semisynthetic (−)-*O*-acetylperhentidine A **54**, and (−)-*O*-acetylperhentidine B **55**, respectively [18]. (−)-Perhentinine **39** and *O*-acetylperhentinine **56** were cytotoxic against KB/VJ300 cells with IC_50_ values of 0.52 and 0.30 μg/mL (μM), respectively, in the studies by Kam et al. [18]. 

(+)-Villalstonine **43** has demonstrated various biological activities including anticancer, antimalarial, and antiamoebic activity. (+)-Villastonine **43** was 1/15th as potent as chloroquine (antimalarial drug) against malaria [45]. It exhibited potent antiplasmodial activity against the multidrug-resistant K1 strain of *P. falciparum* with an IC_50_ value of 0.27 μM [20]. Wright et al. tested this compound for antiamoebic activity against *E. histolytica* [43]. (+)-Villalstonine **43** showed six times less activity (ED_50_ 11.8 μM) than the antiamoebic drug emetine (ED_50_ 2.04 μM). These results also concur with the use of various parts of the *A. angustifolia* plant from ancient times to treat malaria and amoebic dysentery [43]. Moreover, (+)-villalstonine **43** was cytotoxic against KB cells with an ED_50_ value of 11.6 μM [43]. (+)-Villalstonine **43** was also potent against the T9-96 strain of *P. falciparum* with an IC_50_ value of 0.94 μM. It also showed anticancer activity against the HT-29 cell line with an ED_50_ value of 8.0 μM (paclitaxel was the positive control). Also, it was cytotoxic against vincristine-resistant KB/VJ300 cells with an IC_50_ value of 0.42 μg/mL (μM) [18]. On the other hand, the derivative of (+)-villalstonine **43**, villalstonine *N(4)*-oxide **44** was less potent (IC_50_ 10.7 ± 1.9) than (+)-villalstonine **43** itself. The increase in the ionic charge (decrease in lipophilicity) might have reduced the ability of villalstonine *N(4)*-oxide **44** to cross through the cell membranes of red blood cells or the parasites, which if correct, would explain the weaker activity [20]. Villalstonine *N*(4)-oxide **44** is also an antileishmanial bisindole. It was active against promastigotes of *Leishmania mexicana* with an IC_50_ value of 80.3 μM [39]. (+)-Villalstonine **43**-related alkaloids (+)-villalstonidine B **48** and (+)-villalstonidine F **52** were found to be strongly cytotoxic against KB/VJ300 cells with IC_50_ values of 0.35 and 5.64 μg/mL (μM), respectively [18]. (+)-Villalstonidine D **50** exhibited antileishmanial activity. It was active against promastigotes of *L. mexicana* with an IC_50_ value of 120.4 μM in a study from Pan et al. [39]. Another (+)-villalstonine **43**-related alkaloid, (+)-villalstonidine E **51,** demonstrated anticancer and antimalarial activity. It was cytotoxic against the HT-29 cell lines with an ED_50_ value of 6.5 μM and was active against promastigotes of *L. mexicana* with an IC_50_ value of 78 μM in the study from the same group [39]. Many bisindoles are yet to be screened for their activity because of the paucity of material; however, their significant role in future drug discovery should be considered. 

## 4. Biosynthesis

### 4.1. Proposed Biogenetic Pathway toward Angustilongines (**6–9**)

**Figure 1:**
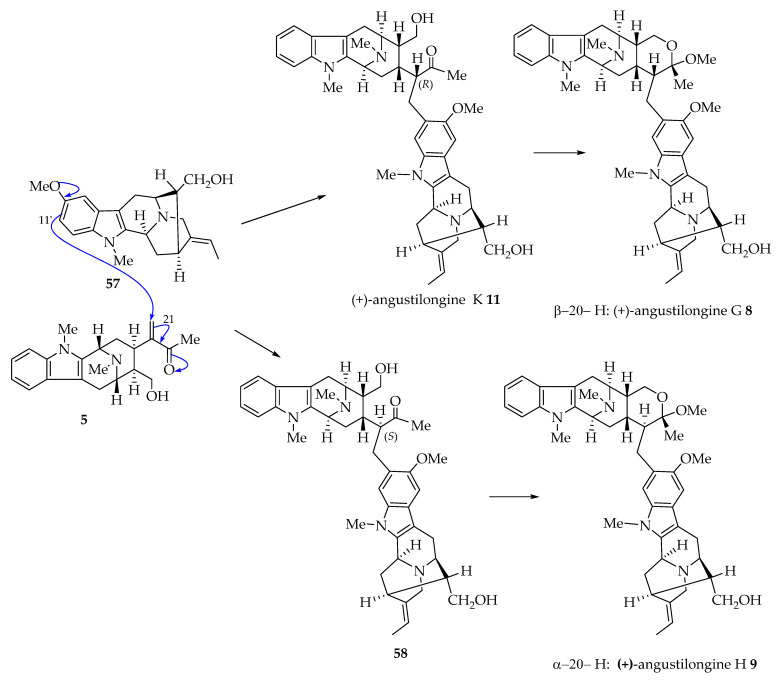
Proposed biogenetic pathway to (+)-angustilongine G **8** and (+)-angustilongine H **9**.

**Figure 2:**
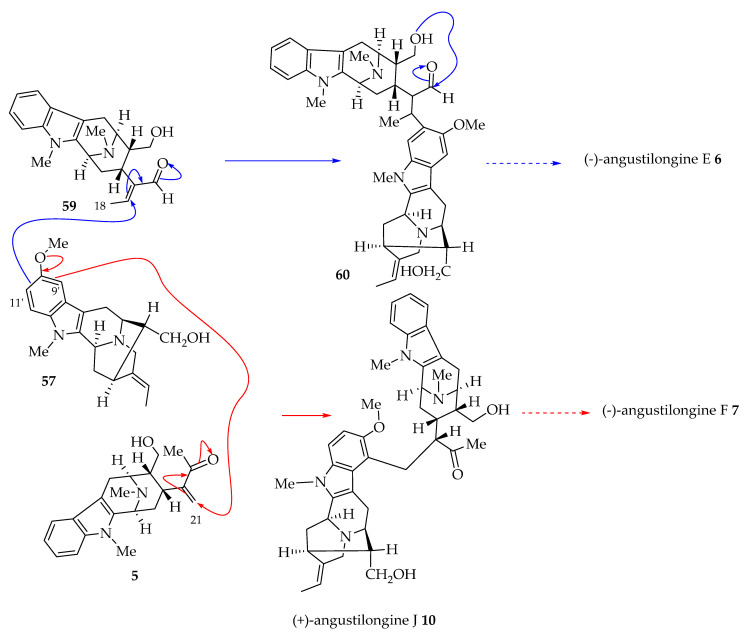
Proposed biogenetic pathway to (−)-angustilongine E **6**, (−)-angustilongine F **7**, and (+)-angustilongine J **10**.

Yeap et al. put forward a plausible biogenetic pathway to the angustilongines [5]. The nucleophilic C-11′ of 10-methoxyaffinisine **57** undergoes conjugate addition onto C-21 of the hypothetical macroline unit **5** to give (+)-angustilongine K **11** and **58** (Figure 1). The (+)-angustilongine K **11** and **58**, after the E-ring closes to the hemiacetal, would give (+)-angustilongine G **8** and (+)-angustilongine H **9**, respectively. Since methanol was used during the extraction of the alkaloids, there could be the possible formation of bisindoles **8** and **9** as artifacts [5]. Similarly, nucleophilic addition of C-9′ of 10-methoxyaffinisine **57** by conjugate addition (Michael reaction) onto C-21 of the macroline unit **5** would give angustilongine J **10**. The E-ring closure of **10** gives angustilongine F **7** (Figure 2). The 10-methoxyaffinisine **57** through its nucleophilic C-11′ carbon atom addition to the C-19 of an *E*-*seco*-talcarpine derivative **59** results in angustilongine E **6** via hydroxy aldehyde **60** [5]. Another likely mechanism is a Friedel–Crafts alkylation of the oxonium ion of corresponding macroline units (cyclization followed by loss of water) to the respective sarpagine units as suggested by Fukuyama to furnish angustilongines (mechanism not shown for angustilongines **6**–**11** see Section 4.4 with representative examples of (+)-macralstonine **24** and (+)-lumutinine A **18**) [6].

### 4.2. Plausible Biogenetic Pathway to (-)-Lumusidines A-D (**14–17**)

**Figure 3:**
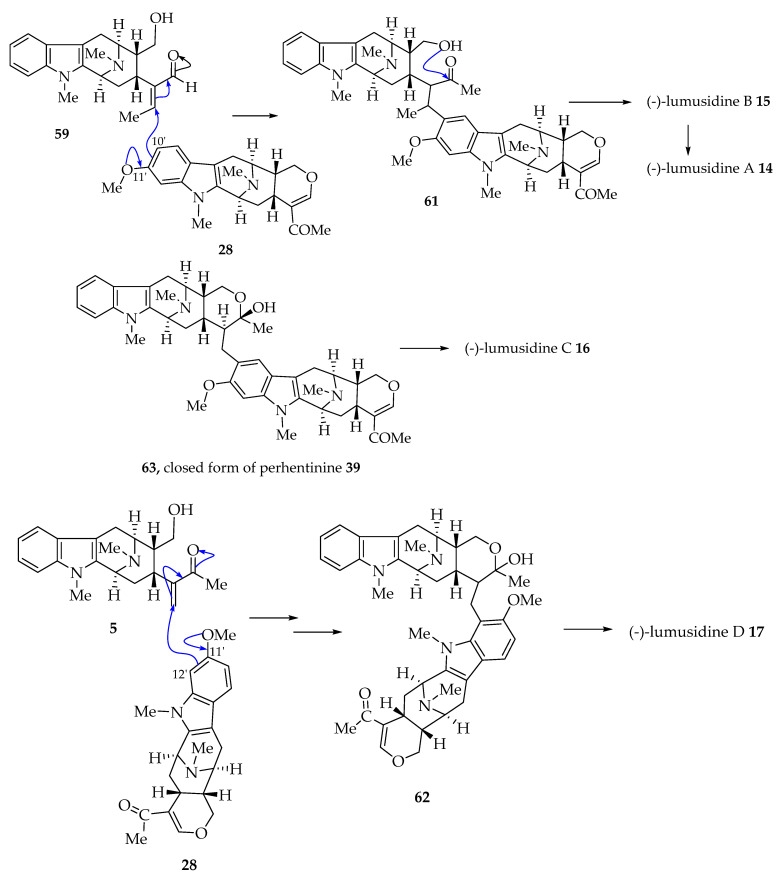
Putative biogenetic pathway to (-)-lumusidines A–D (**14**–**17**).

The conjugate addition of the C-10′ carbon atom of alstophylline **28** by a Michael reaction onto the α, β unsaturated aldehyde **59** (E-ring opened talcarpine derivative) could potentially give hydroxy ketone bisindole alkaloid **61** (Figure 3). Alcohol **61** on subsequent closing of the E-ring to form a hemiacetal gives (-)-lumusidine B **15**, which after dehydration gives (-)-lumusidine A **14**. Similarly, the C-12′ carbon atom of alstophylline monomer **28** could add to the α, β unsaturated macroline counterpart **5** to furnish a hydroxy ketone (not shown). The cyclization of the hydroxy ketone in a similar way to give a hemiacetal **62** would follow. Subsequently, the dehydration of hemiacetal **62** would likely form lumusidine D **17**. The lumusidine C **16** is assumed to be an artifact formed from hemiacetal **63** (a closed form of (-)-perhentinine **39**) as ethanol was used during the extraction [18]. However, again this biosynthetic pathway could occur just as likely via a Friedel–Crafts alkylation of the oxonium ion of **59** (cyclization followed by loss of water) to the olefin of precursor **28** (see Section 4.4 with representative examples of (+)-macralstonine **24** and (+)-lumutinine A **18**) [6].

### 4.3. Proposed Biogenetic Pathway to Lumutinines A–D (**16–19**)

**Figure 4:**
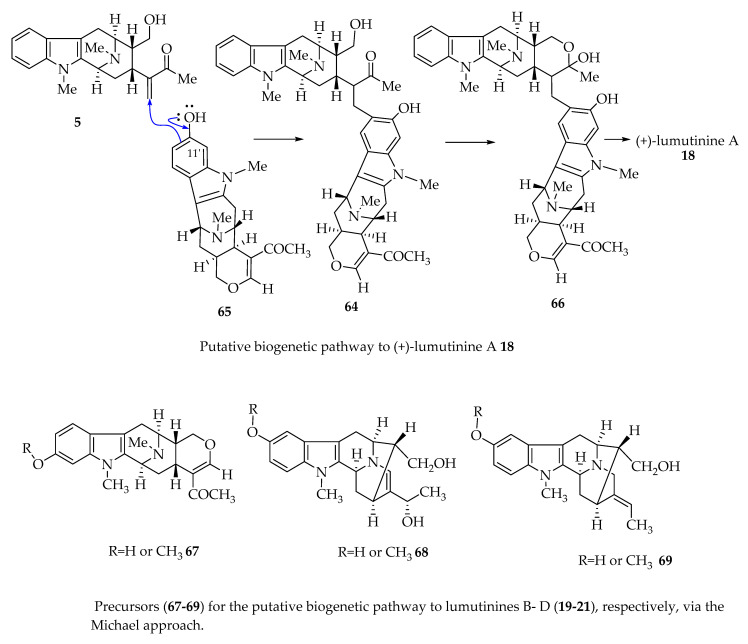
Putative biogenetic pathway to lumutinines A–D (**16**–**19**).

The hydroxy ketone **64** could be formed by Michael addition of the C-10’ carbon atom of alstophylline **65** onto the olefinic moiety of macroline **5** (Figure 4) [26]. The cyclization of the hydroxy ketone would give the hemiketal **66,** which on ketalization furnishes (+)-lumutinine A **18**. Likewise, Michael addition by C-12’ of **67**, C-9’ of **68,** and C-11’ of **69** (Figure 4) onto macroline **5** yields (-)-lumutinine B **19**, (+)-lumutinine C **20**, and (+)-lumutinine D **21,** respectively [26]. Again, the Friedel–Crafts alkylation of the oxonium ion of **65** (cyclization followed by loss of water) is just as likely (see Section 4.4 with representative examples of (+)-macralstonine **24** and (+)-lumutinine A **18**) [6].

### 4.4. Possible Alternative Mechanism of Bisindole Formation of (+)-Lumutinine A **16** and (+)-Macralstonine **24** as Representative Examples via a Friedel–Crafts Alkylation Process as Suggested by Fukuyama

**Figure 5:**
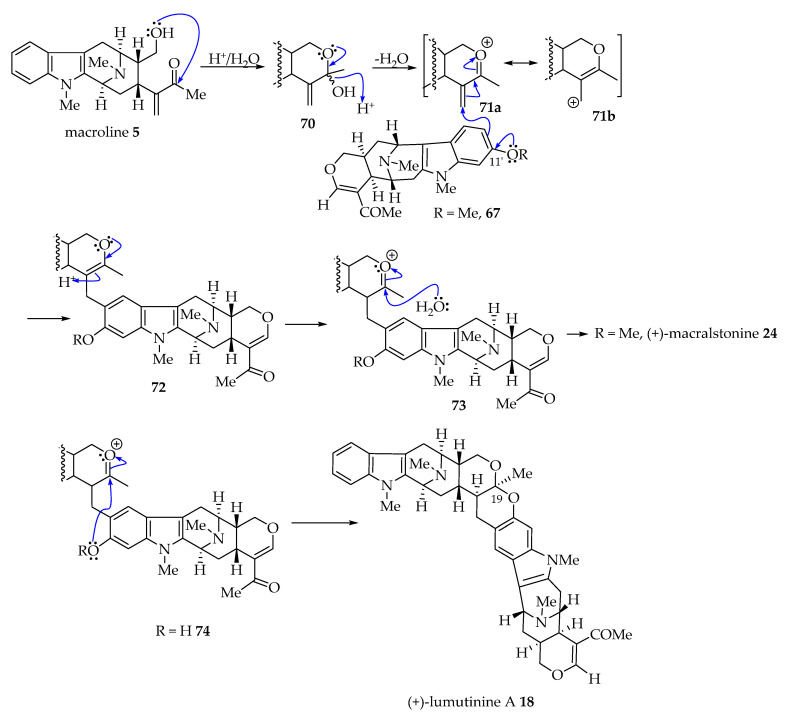
Possible alternative mechanism of bisindole formation via a Friedel–Crafts alkylation process for (+)-macralstonine **24** and (+)-lumutinine A **18**, respectively, as representative examples.

In addition to the Michael addition process described above, there is another potential mechanism for the coupling of ring-A oxygenated macroline-type alkaloids with macroline **5** that involves a Friedel–Crafts alkylation process stabilized by an oxonium ion [6]. As shown in the figure below (Figure 5), an acid-catalyzed intramolecular cyclization of macroline **5** by nucleophilic attack of the C-19 hydroxyl function onto the carbonyl carbon atom in 1, 2-addition fashion would furnish the cyclic hemiacetal **70**. The loss of a water molecule would generate an oxonium ion species (**71a** or **71b)**. At this stage, an electrophilic attack of the electron-rich aromatic ring of 11-methoxy macroline **67** via the C-10 carbon atom onto the olefin in **71a** would generate a cyclic enol ether **72**. This cyclic hemiacetal could undergo isomerization, generating an oxonium ion intermediate **73** only to be attacked by water to generate the cyclic ether (as in macralstonine **24**). Alternatively, the phenolic hydroxy group at the C-10 carbon atom in 10-hydroxy macroline (see **74**) could attack the oxonium ion to form a cyclic ketal (as in lumutinine A **16**). This type of mechanism was suggested by Fukuyama for some bisindoles isolated from *Alstonia* species [6].

### 4.5. Proposed Biogenetic Pathway to (-)-Macrocarpamine **31**

**Figure 6:**
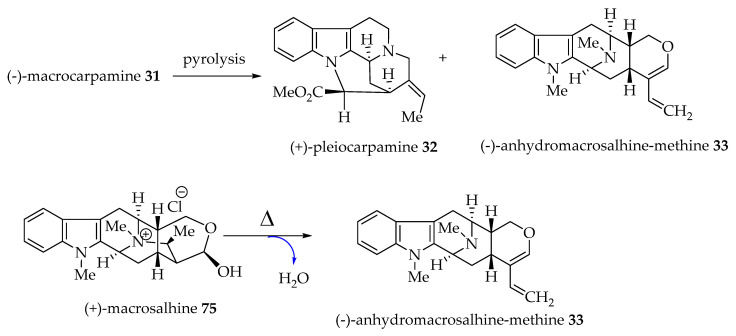
Pyrolysis of (-)-macrocarpamine **31** and macrosalhine **75** to generate key olefin **33**.

(-)-Macrocarpamine **31**, a bisindole alkaloid comprised of two *Alstonia* monomeric units (+)-pleiocarpamine **32** and (-)-anhydromacrosalhine-methine **33,** was proposed to originate from (-)-anhydromacrosalhine-methine **33** and (+)-pleiocarpamine **32** by Hesse, Schmid et al. [46]. (-)-Anhydromacrosalhine-methine **33** and (+)-pleiocarpamine **32** are the degradation products of (-)-macrocarpamine **31**. (-)-Anhydromacrosalhine-methine **33** was also obtained by dehydration of (+)-macrosalhine **75** in the study by Khan et al. (Figure 6) [6,47].

## 5. Synthesis of Representative Monomeric Units of Bisindole Alkaloids from *Alstonia* Species Discussed Herein, Required for Bisindole Synthesis

### 5.1. The Total Synthesis of (±)-Pleiocarpamine **32**

**Scheme 1:**
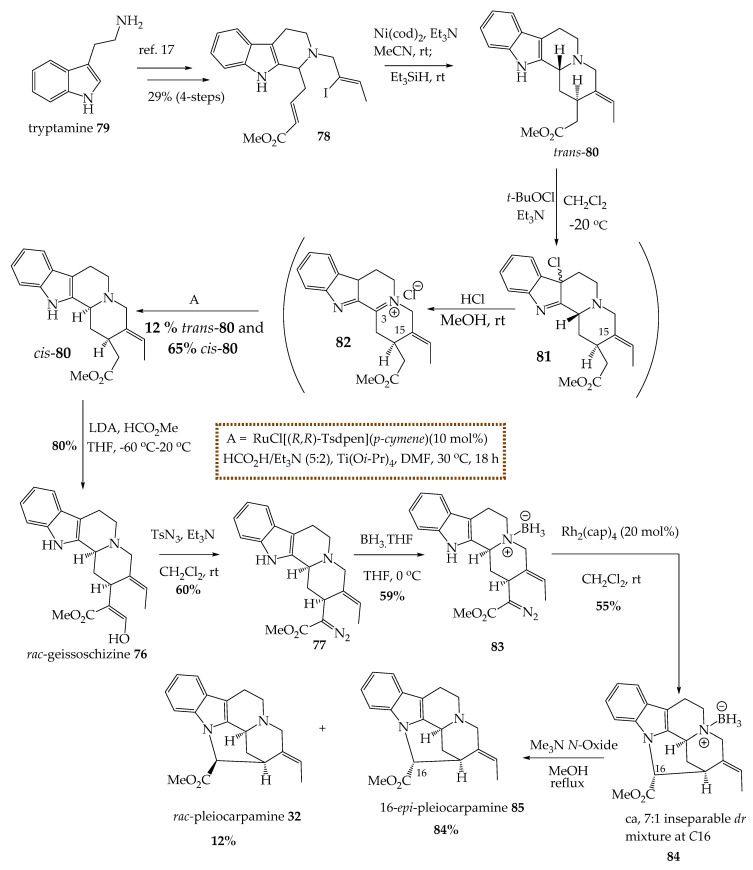
Synthesis of *rac*-pleiocarpamine **32** and 16-*ep*i-pleiocarpamine **85** by Sato et al.

(+)-Pleiocarpamine **32** has been isolated from various species of *Alstonia* [6,32,36,48,49,50,51]. The unprecedented (+)-bipleiophylline (not shown) was found to contain two pleiocarpamine **32** units connected by utilizing an aromatic spacer (pyrocatechuic acid). The homo-dimer (+) bipleiophylline was potentially active against drug-sensitive, vincristine-resistant human KB and Jurkat cells, while its monomeric precursor (+)-pleiocarpamine **32** was not very active [52]. These findings again demonstrate the increased activity of dimeric alkaloids as compared to their monomeric counterparts. The (+)-pleiocarpamine **32** was thought to be formed biosynthetically by a ring-closing reaction between *N*1 and *C*16 of geissoschizine **76** [17]. Recently Sato et al. reported the total synthesis of the *C*-mavacurine type indole alkaloid, (±)-pleocarpamine **32** via a biomimetic synthesis [17]. A critical step, the direct cyclization between *N*1 and *C*16, was thought to be challenging because of the strain generated in the corynanthe pentacyclic framework. This may likely be the reason for the time it took to achieve the total synthesis of pleiocarpamine **32** by many groups since its isolation. In fact, (±)-2,7-dihydropleiocarpamine (not shown) was synthesized in 1993 by Jiménez et al. [53]. However, Sato et al. successfully cyclized the strained structure of **77** by a carbene *N*-H insertion reaction between the *N*1 and *C*16 bonds (Scheme 1) [17]. 

The ester **78**, was prepared in four steps from tryptamine **79** in 29% overall yield [17,54]. The subsequent reductive cyclization of ester **78** onto *cis*-ester **80** was carried out using Ni(cod)_2_. However, at this step, the desired cyclized *cis*-ester **80** was formed as a minor product (31%) with the undesired *trans*-ester **80** (55%) predominating [17]. The undesired *trans*-ester **80** was first treated with *tert*-butyl hypochlorite and triethylamine at –20 °C to obtain intermediate **81**, which on further reaction with hydrochloric acid in methanol at room temperature furnished iminium intermediate **82** [17]. Sato et al. screened several reducing agents and reaction conditions to reduce the iminium intermediate **82** into the *cis*-ester **80.** The optimized condition for the reduction of the iminium intermediate employed by Sato et al. was a Noyori catalyst and formic acid/triethylamine (5:2) mixture with titanium *tetra*-isopropoxide (at 30 °C) to yield the desired *cis*-ester **80** as a major product (*cis/trans* ratio 5:3.1) [17]. The *cis*-ester **83** was converted into (±)-geissoschizine **76** in 80% yield using an excess of lithium diisopropylamine and methyl formate. The treatment of (±)-geissoschizine **79** with tosyl azide and triethylamine furnished diazo compound **77** in 60% yield [17].

While exploring a suitable catalyst for the metal carbenoid cyclization reaction, the Rh_2_(cap)_4_ and Rh_2_(esp)_2_ catalyzed reactions lead to decomposition of substrate **77** [17]. Only Rh_2_(OAc)_4_ furnished the distinguishable but unanticipated product (not shown) via a *D*-ring expansion reaction [17]. At room temperature, no reaction happened while employing the rhodium complex (PPh_3_)_3_RhCl or the copper catalyst Cu(MeCN).BF_4_ [17]. Moreover, the use of the JohnPhosAu(MeCN)SbF_6_ catalyst was also unsuccessful because it formed the *D*-ring expanded product (not shown) with β-hydride elimination (not shown) [17]. Sato et al. predicted that a modification of the lone pair on the *N*(4) of the compound **77** would form a *cis*-quinolizidine ring making cyclization more favorable by decreasing the distance between *N*1 and *C*16. This was the key to the first total synthesis of (±)-pleiocarpamine **32**. Thus, substrate **77** was modified to borane salt **83** by using the BH_3_–THF complex to react at the *N*_b_ nitrogen atom [17]. Finally, cyclization via a carbene N–H insertion was accomplished by treatment of cyclization precursor **83** with Rh_2_(cap)_4_ (20 mol %) at room temperature, and the products so obtained were an inseparable diastereomeric mixture **84** at *C*_16_ (ca,7:1) in 55% yield [17]. To remove the borane protecting group, the diastereomeric mixture **84** was refluxed with trimethylamine *N*-oxide in methanol, which furnished (±)-pleiocarpamine **32** and epi-pleiocarpamine **85** [17].

### 5.2. Biosynthetic Relations among the Sarpagine/Macroline/Ajmaline Family of Alkaloids

**Figure 7:**
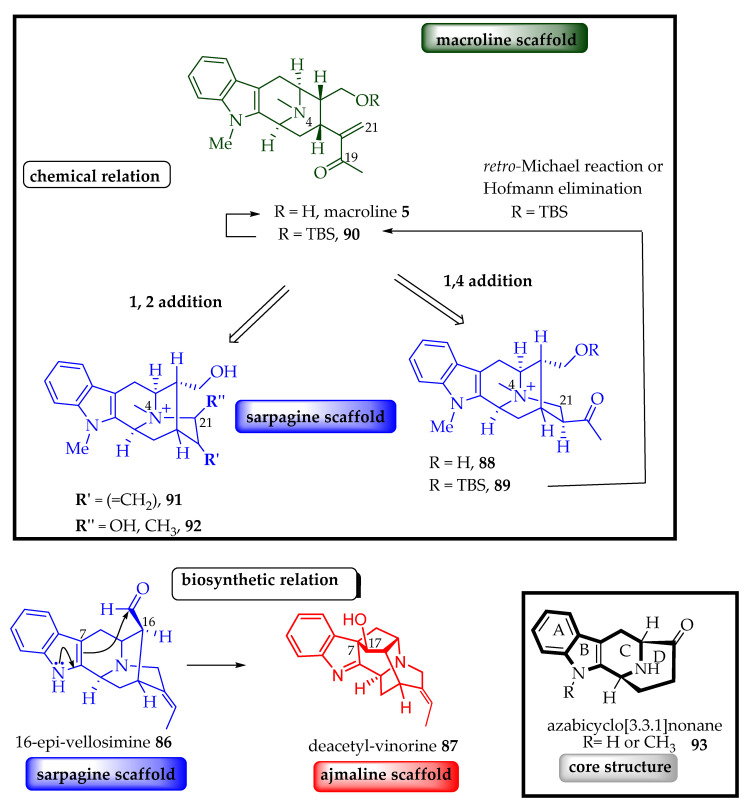
Abbreviated chemical and biosynthetic relations among the sarpagine/macroline/ajmaline family of alkaloids.

Sarpagine, macroline, and ajmaline indole alkaloids are biosynthetically and structurally related (Figure 7) [13]. We herein follow the biogenetic numbering system of LeMen and Taylor to discuss their potential formation [55]. The sarpagine series consists of attachment of the C-21 carbon atom to the *N*(4) bond, while the macroline series lack this bond. The stereocenters in the sarpagine alkaloids at C-3 (*S*), C-5 (*R*), C-15 (*R*), and C-16 (*R*) are identical to the macroline series at the respective carbon atoms. The ajmaline series also contain a C-7 and C-17 carbon atom linkage. In comparison with the ajmaline series, the sarpagine family at C-16 is *R* but antipodal in the ajmaline series. Both the sarpagine and the ajmaline series consist of the characteristic quinuclidine ring and the C-5 and C-16 carbon atom linkage. Sarpagine and ajmaline alkaloids both contain the characteristic C-21 and *N*(4) bond [13,56]. Stӧckigt et al. reported the conversion of 16-epi-vellosimine **86** (sarpagine) into the vinorine **87** (ajmaline) structure (Figure 7) by employing the acetyl CoA-dependent vinorine synthase enzyme [57,58,59,60]. The macroline and sarpagine alkaloids are biosynthetically related, as shown in Figure 7. Either of the β-keto quaternary ammonium intermediates **88** or **89** can be furnished by 1, 4 Michael addition of the *N*(4) nitrogen atom of macroline onto the α, β-unsaturated carbonyl system **5** or **90** at C-21. Similarly, α-hydroxy quaternary ammonium compounds **91** and **92** can be formed by direct 1, 2 addition of the *N*(4) nitrogen atom to the C-19 carbonyl carbon of **5** and **90** [13]. In contrast, the *retro*-Michael reaction of TBS protected intermediate **89,** followed by removal of the silyl protecting group converted sarpagine **89** into the macroline unit **5** in the studies from LeQuesne et al. [61] and Cook et al. [62]. The sarpagine/macroline/ajmaline group of indole alkaloids have been isolated principally from higher plants, including the Apocynaceae family and the genera *Alstonia* and *Rauwolfia* [13]. The intriguing molecular complexity, interconversions, and their divergent biological activity prompted the successful syntheses of several of these indole alkaloids [7,63] and bisindole alkaloids [45,64,65] as mentioned earlier.

### 5.3. Synthesis of Monomeric Macroline Units Employed for the Synthesis of Bisindole Alkaloids

#### 5.3.1. An Improved Total Synthesis of (-)-Alstophylline **28**

**Scheme 2:**
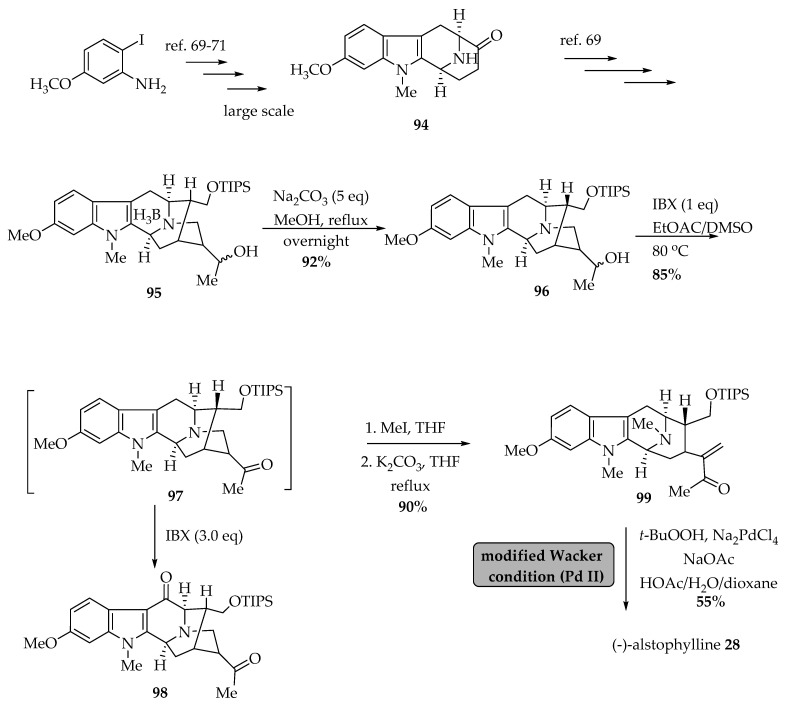
An improved total synthesis of (-)-alstophylline **28** using modified Wacker (Pd II) conditions.

Liao et al. [66] improved the synthesis of (-)-Alstophylline **28** which, has been isolated from various *Alstonia* species including *A. angustifolia* [36], *A. glabriflora* Mgf. [31], and *A. macrophylla* [30,67,68]. Liu et al. accomplished a large-scale enantiospecific total synthesis of intermediates, which later resulted in the total synthesis of (-)-alstophylline **28** [69]. Later, Liao et al. improved the total synthesis of (-)-alstophylline **28** employing modified Wacker reaction conditions, which provided a faster route for synthesis of macroline-related indole alkaloids (Scheme 2) [66]. The 6-methoxy tetracyclic ketone **94** was synthesized employing the method using the published procedures [69,70,71], which later was converted into the *N*_b_-BH_3_ adduct **95** using the method developed by Liu et al. earlier [69]. The *N*_b_-BH_3_ adduct **95** so formed was refluxed with sodium bicarbonate in methanol overnight to get alcohol **96** in 92% yield. One equivalent of the 2-iodozybenzoic acid (IBX) converted (at high temperature) the alcohol functional group in **96** into the ketone **97** in 85% yield. The addition of three more equivalents of IBX at reflux permitted the formation of the new alkaloid 6-oxoalstophylline **98**. The *N*_b_-quaternization of ketone **97** was carried out using MeI/THF, which was followed by a *retro*-Michael reaction in the presence of K_2_CO_3_/THF to furnish *α*,*β*-unsaturated ketone **99** in 90% yield. Finally, the (-)-alstophylline **28** was obtained using the Wacker-related process (Pd II) modified by Tsuji et al. [72] and Cook et al. [66] from ketone **99** in 55% yield. Importantly, the modified Wacker-related process is also an improved route to several indole alkaloids with the enone system including alstonerine **100** [6]. In addition, the key oxidation reaction provides a shorter route from **97** to 6-oxoalstophylline **98**.

#### 5.3.2. Enantiospecific Total Synthesis of (-)-Anhydromacrosalhine-methine **33** via the Asymmetric Pictet–Spengler Reaction

**Scheme 3:**
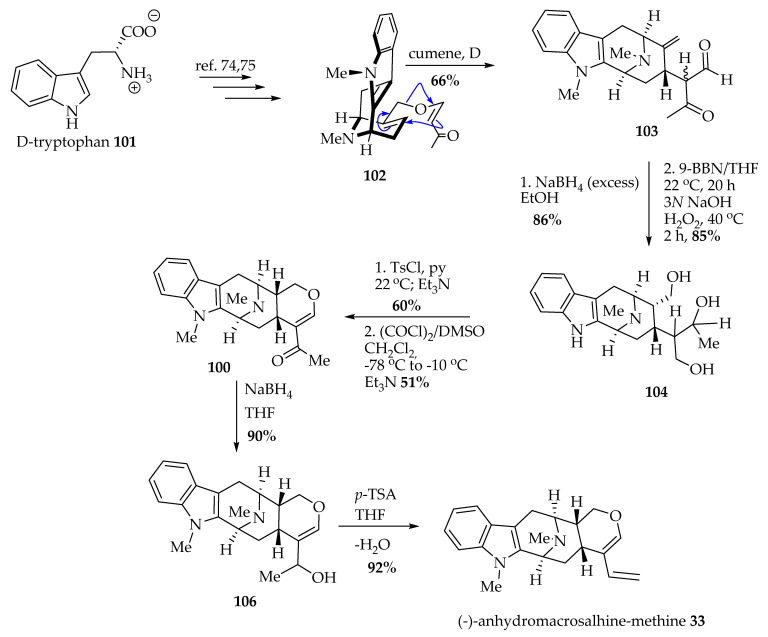
Enantiospecific total synthesis of (-)-anhydromacrosalhine-methine **33** via the asymmetric Pictet–Spengler reaction.

In a partial synthesis, Gan et al. [73] prepared (-)-anhydromacrosalhine-methine **33** via degradation of natural (+)-ajmaline by following the improved procedure of Sakai et al. [73]. In addition to the semisynthesis of (-)-anhydromacrosalhine-methine **33** for comparison purposes, Gan et al. also accomplished the total synthesis of **33** starting from D-tryptophan **101** via the asymmetric Pictet–Spengler reaction (>98% ee) (Scheme 3) [44]. The enone ether **102** was synthesized employing the procedures developed in Milwaukee [74,75]. The β-dicarbonyl compound **103** was prepared in 66% yield from a stereoselective (α-face) Claisen rearrangement of olefin **102** at high temperature (175 °C) [44]. The reduction of the β-dicarbonyl compound **103** that resulted with NaBH_4_ provided the diol (not shown), which subsequently underwent a stereospecific hydroboration–oxidation reaction and was converted into triol **104**. (-)-Alstonerine **100** was furnished by the regioselective cyclization reaction of the monotosylate of triol **104**, which was followed by the improved Swern oxidation in 51% yield, accompanied by dihydroalstonerine **105** (not shown in Scheme 3) in 31% yield [44,75]. (-)-Alstonerine **100**, upon reduction with NaBH_4_, furnished allylic alcohol **106** in 90% yield, which later was dehydrated in the presence of *p*-toluenesulfonic acid to afford (-)-anhydromacrosalhine-methine **33** in 92% yield [44].

#### 5.3.3. Improved Synthesis of (+)-Macroline **5**

Alkaloids such as (-)-alstonerine **100**, (+)-alstonisidine **3**, and (-)-alstophylline **28** are believed to originate from (+)-macroline **5** as their biogenetic precursor unit; however, the isolation of (+)-macroline **5** has not yet been reported [6]. The synthesis of (+)-macroline 5 was improved by Liao et al. [76], whereas the first biomimetic synthesis of (+)-macroline **5** was carried out by LeQuesne et al. in 1980 [61], following the procedure of Gorman et al. [77]. Neukomm et al. elucidated the absolute configuration of macroline **5** with X-ray crystallographic data [78]. Bi et al. accomplished the enantiospecific total synthesis of (+)-macroline **5** [79], which was indistinguishable from the spectral data compared to the natural one [80]. The formal synthesis of (+)-macroline **5** was also reported by Kwon utilizing a phosphine-catalyzed annulation process (not shown) [81]. 

**Scheme 4:**
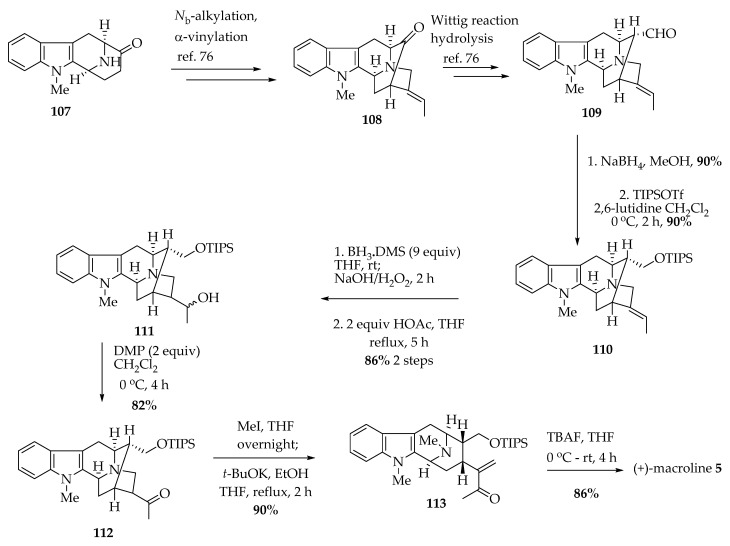
Improved synthesis of (+)-macroline **5**.

Later, Liao et al. improved the total synthesis of (+)-macroline **5** in higher overall yield and fewer steps. The tetracyclic ketone **107**, obtained on the large scale by following the procedure developed in Milwaukee, which underwent *N*_b_-alkylation with (*Z*)-1-bromo-2-iodo-2-butene (not shown), was followed by a palladium catalyzed stereospecific α-vinylation to provide pentacyclic ketone **108** (Scheme 4) [76]. The ketone **108** underwent the usual one-carbon homologation via a Wittig olefination, and this was followed by acidic hydrolysis to obtain the pentacyclic aldehyde **109**. The aldehyde functional group in **109** was reduced using sodium borohydride to provide the first enantiospecific synthesis of (+)-affinisine (not shown) in 90% yield. Then triisopropylsilyl (TIPS) triflate and 2,6-lutidine were used to protect the primary alcohol of (+)-affinisine as a TIPS ether **110** in 90% yield. The TIPS ether **110** was employed for the hydroboration–oxidation reaction, which furnished the desired secondary alcohol **111** in 86% yield. The alcohol **111**, so formed, was oxidized to a ketone **112** using the Dess-Martin periodinane reagent in 82% yield. The ketone **112** was quaternized at the *N*_b_-nitrogen atom by treatment with iodomethane in THF at 0 °C. The stable macroline equivalent **113** was obtained after treatment of *N*_b-_methyl iodide salt with potassium *tert*-butoxide in ethanol, after which it was treated with tetrabutylammonium fluoride to furnish macroline **5** in **86%** yield (99% ee) after removal of the TIPS group [76].

#### 5.3.4. Stereospecific Access to the Macroline Core **5**

**Scheme 5:**
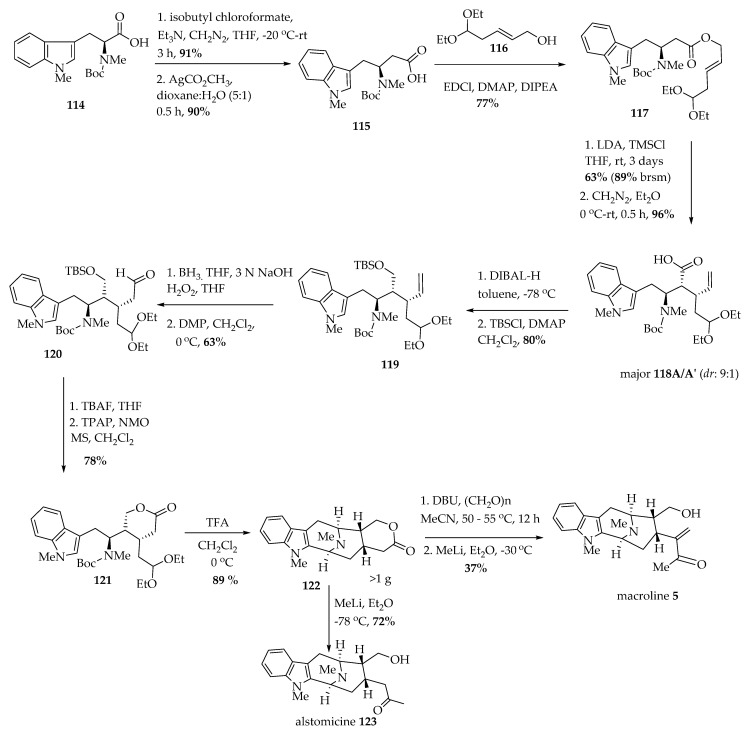
Stereoselective total synthesis of macroline **5** and alstomicine **123**.

Recently, Kadam et al. developed another route to the pentacyclic core of macroline **5**-related alkaloids. Starting from commercially available L-tryptophan (not shown), the core architecture of macroline **5** was accessed via an Ireland–Claisen rearrangement (ICR)/Pictet–Spengler cyclization protocol (Scheme 5) [82]. The L-tryptophan derivative **114** was subjected to Arndt–Eistert conditions to implement a one-carbon homologation to furnish acid **115**. The acid **115** was subsequently coupled with allylic alcohol **116** under standard amide coupling conditions to furnish the allylic ester **117**, the substrate for the Ireland–Claisen rearrangement. The allylic ester **117** produced the corresponding carboxylic acids (not shown) under ICR as an inseparable mixture that was converted into the corresponding esters **118A/A’** by treating the ICR mixture. The esters were isolated in a 9:1 ratio of diastereomers that was separable. The ester function in **118A** was reduced by DIBAL-H, and the so formed primary alcohol was protected with a TBS group to provide silyl ether **119**. The olefin **119** was subjected to a hydroboration–oxidation sequence and this was followed by oxidation of the so formed alcohol with Dess-Martin periodinane to furnish aldehyde **120**. Upon desilylation with TBAF, the alcohol reacted intramolecularly with the proximate aldehyde function forming the corresponding hemiacetal (not shown). Subsequent *tetra*-propylammonium perruthenate (TPAP)/*N*-methylmorpholine *N*-oxide (NMO) mediated oxidation of the hemiacetal furnished δ-lactone **121**. Deprotection of the Boc protecting group and hydrolysis of the acetal was done under acidic conditions with TFA setting up the conditions for an intramolecular Pictet–Spengler cyclization that furnished the pentacyclic core structure **122** of the macroline alkaloids. The exomethylene was introduced by reacting **122** with the formaldehyde equivalent in the presence of 1,8-*di*-azabicyclo[5.4.0]undec-7-ene (DBU). This was followed by the addition of MeLi to furnish macroline **5**. On the other hand, alstomicine **123** was achieved by treating the lactone **122** with MeLi [82].

#### 5.3.5. Enantiomeric Total Synthesis of (+)-*N*_a_-Methylsarpagine **124**

**Scheme 6:**
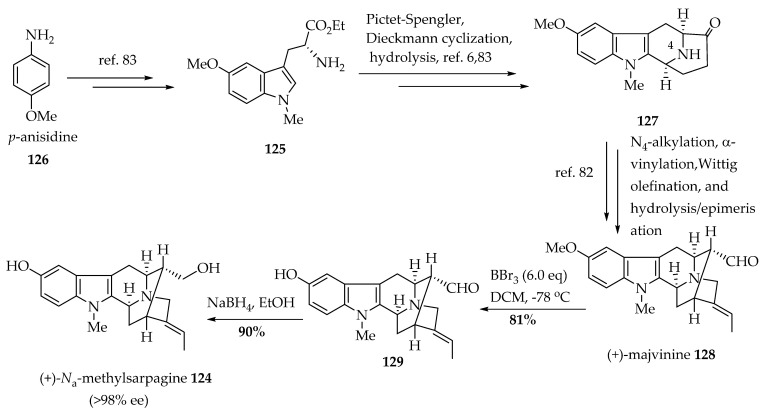
Enantiomeric total synthesis of (+)-majvinine **128** and (+)-*N*_a_-methylsarpagine **124**.

The alkaloid (+)-*N*_a_-methylsarpagine **124** was isolated from the bark of *A. spectabilis* [31,41], which is present as a monomeric unit in the (+)-macralstonidine **23** bisindole. The first total synthesis of (+)-*N*_a_-methylsarpagine **124** was accomplished by Zhao et al. [83,84]. The 5-methoxy-*N*_a_-methyl d-(+)-tryptophan ethyl ester **125** was prepared on a large scale starting from commercially available *p*-anisidine **126** (Scheme 6). The 5-methoxy tetracyclic ketone **127** was furnished in high overall yield from ester **125** employing chemical conversions such as, *N*_b_-benzylation, the asymmetric Pictet–Spengler reaction, diastereospecific Dieckmann cyclization, decarboxylation reaction, and catalytic debenzylation [6,83]. Furthermore, 5-methoxy tetracyclic ketone **127** upon further usual transformations including *N*_b_-alkylation, the Wittig olefination, hydrolysis/epimerization gave majvinine **128** in 98% ee [83,84]. The 10-hydroxy indole **129** was furnished after the treatment of C-10 methoxy intermediate (+)-majvinine **128** employing six equivalents of BBr_3_ in DCM at −78 °C. The subsequent reduction of the aldehyde group in intermediate **129** was effectively carried out using sodium borohydride in ethanol to furnish (+)-*N*_a_-methylsarpagine **124** in 90% yield [83].

## 6. The Synthesis of Bisindole Alkaloids

### 6.1. Biomimetic Partial Synthesis of (-)-Alstonisidine **3**

(-)-Alstonisidine **3** contains a (+)-quebrachidine **4** (ajmaline-type) and a (+)-macroline **5** piece as monomeric units. Both monomeric alkaloids were employed in the biomimetic partial synthesis of (-)-alstonisidine **3** by LeQuesne, Burke, and Cook [85]. The (+)-macroline **5** used in this partial synthesis was obtained after the degradation of villamine (not shown) by the method of Schmid et al. [80]. 

**Scheme 7:**
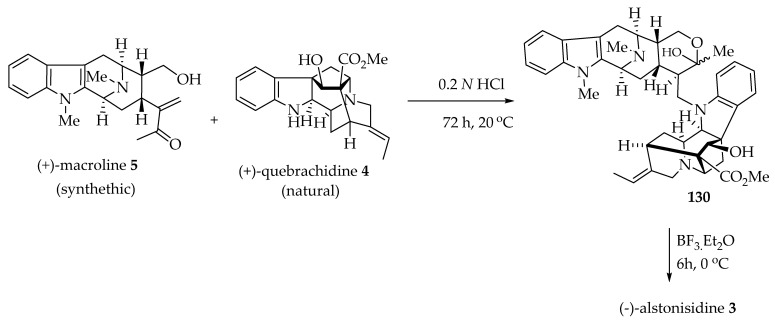
Biomimetic partial synthesis of (-)-alstonisidine **3**.

The hemiketal **130** was formed after the Michael reaction between the *N*_a_-H function of a (+)-quebrachidine **4** and (+)-macroline **5** in the presence of 0.2 *N* HCl at 20 °C for 3 days (Scheme 7). The hemiketal **130** underwent an electrophilic substitution reaction (*ortho* to the amino group of (+)-quebrachidine **4**) after the treatment with BF_3_ etherate at low temperature (0 °C) to furnish (-)-alstonisidine **3.** The spectral data of (-)-alstonisidine **3** were identical to those of naturally isolated (-)-alstonisidine **3** [37,51]. 

### 6.2. Biomimetic Partial Synthesis of (+)-Dispegatrine **131**

**Scheme 8:**
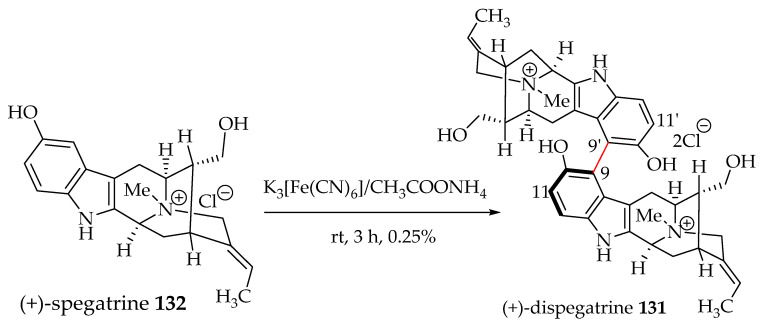
Biomimetic partial synthesis of (+)-dispegatrine **131** by oxidative phenolic coupling by Yu et al. [86].

Although the focus of this review is bisindoles from *Alstonia* species, dispegatrine **131**, a hypotensive bisindole, is comprised of two sarpagine units. It is discussed here because some of the monomers such as sarpagine are commonly found in many *Alstonia* species. In the biomimetic partial synthesis of (+)-dispegatrine **131** by Yu et al., the monomer spegatrine **132** was subjected to an oxidative phenolic coupling (Scheme 8). Although the yield of this coupling was very poor, it served as proof of the concept that dispegatrine **131** may actually originate biosynthetically via oxidative phenolic coupling, although the chemical yield was 0.25%. This partial synthesis confirmed the structure of (+)-dispegatrine **131** [86]. However, no other diastereomer was observed, and the axial chirality across C9-C9′ could not be confirmed. In addition, due to the lack of X-ray crystals, the isolation chemists could not assign the important axial chirality at C9-C9′ [86]. 

#### Enantiospecific Total Synthesis of (+)-dispegatrine **131** in >98% ee

**Scheme 9:**
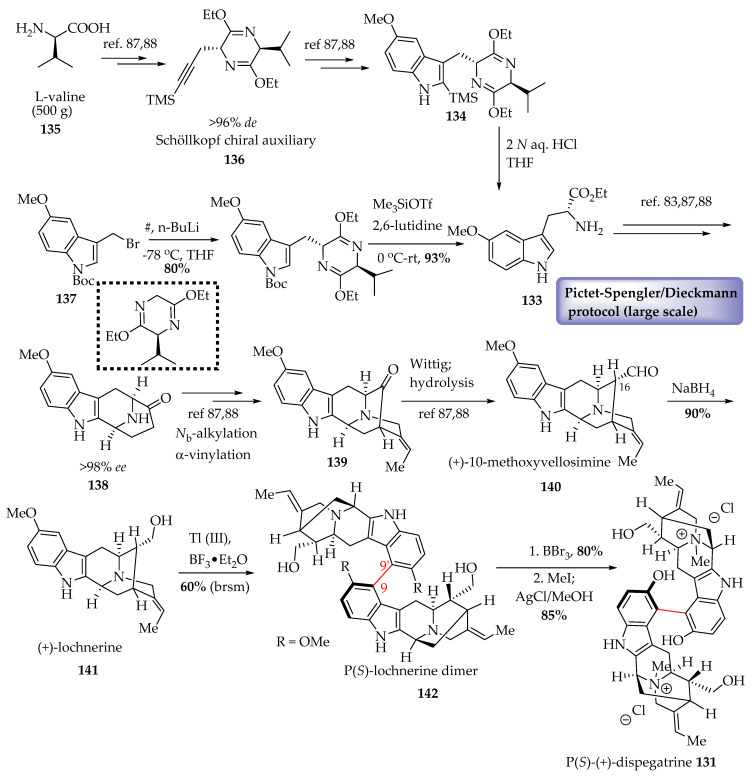
The biomimetic total synthesis of the natural P(*S*)-atropodiastereomer of (+)-dispegatrine **131**.

It was suggested that internal chiral induction was responsible for the atroposelectivity, which was exploited as an advantage in the first enantiospecific and convergent total synthesis of (+)-dispegatrine **131** from spegatrine **132** by Edwankar et al. [87,88]. The total synthesis of dispegatrine **131** required the 5-methoxy tryptophan unit **133**, which was accessed via the general strategy for the synthesis of ring-A oxygenated D-tryptophan unit **134** previously developed in Milwaukee (Scheme 9). L-Valine **135** served as the source of chirality in the Schöllkopf chiral auxiliary **136** and was employed in a large-scale reaction (500 g). After obtaining the required chiral starting material, D-tryptophan ethyl ester **133** (which could also be obtained from the Boc protected 5-methoxy indole **137** (Scheme 9)), the large-scale Pictet–Spengler/Dieckmann protocol developed in Milwaukee was employed to gain access to the key tetracyclic ketone **138** in excellent yield and >98% enantiomeric excess. This tetracyclic ketone **138** furnished the sarpagine system **139** after *N*_b_-alkylation and α-vinylation, both of which could also be performed on large scales. The pentacyclic ketone **139** containing the sarpagine architecture was further functionalized. A one-carbon homologation via a Wittig olefination, followed by hydrolysis under acidic conditions, furnished 10-methoxy vellosimine **140** for the first time (>98% ee). Reduction of the C-16 formyl function with NaBH_4_ furnished (+)-lochnerine **141** in excellent yield. A thallium (III)-mediated oxidative coupling facilitated by borane trifluoride diethyl etherate as the Lewis acid provided the dimerized lochnerine **142** as the P(*S*)-atropodiastereomer. The absolute configuration was confirmed by X-ray analysis with a MoKα source at low temperature. At the conclusion of this total synthesis, the final assault, demethylation of the two methoxy groups followed by quaternization of the *N*_b_-nitrogen atoms was performed. Treatment of the lochnerine dimer **142** with boron tribromide etherate furnished the bis-phenolic dimer (not shown) in 80% yield. Quaternization was executed by treating the tertiary amine functions with excess iodomethane, and this was followed by exchange of iodide with chloride as the counter anion with the help of a AgCl additive. This furnished the first and biomimetic total synthesis of the antihypertensive bisindole, the P(*S*)-atropodiastereomer of (+)-dispegatrine **131** [87,88].

### 6.3. Biomimetic Total Synthesis of (+)-Macralstonidine **23**

**Scheme 10:**
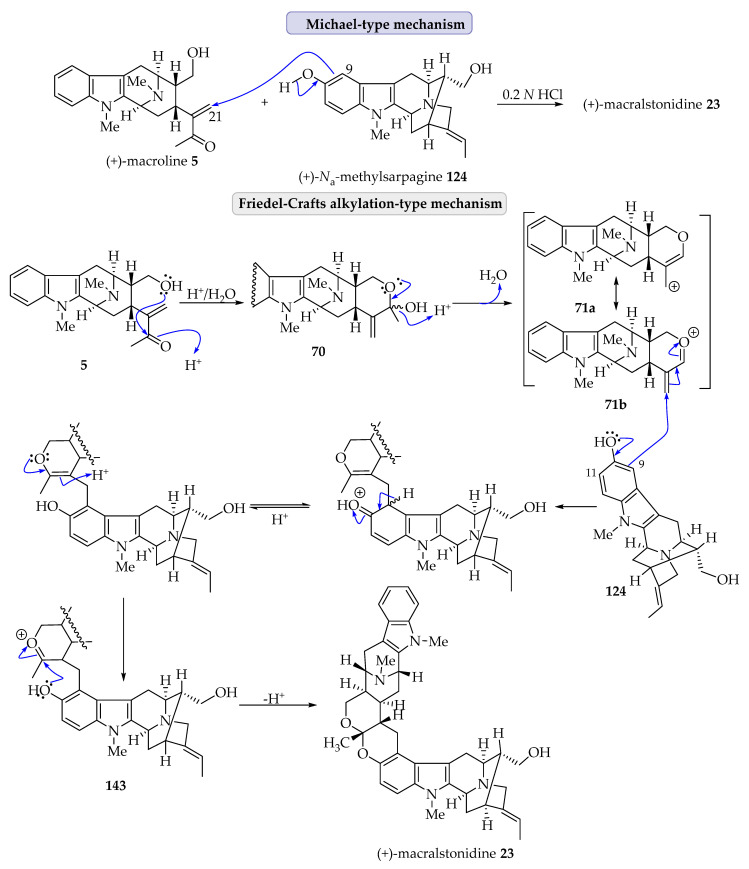
Biomimetic total synthesis of macralstonidine **23** by the condensation of macroline **5** with *N*_a_-methyl sarpagine **124** via two possible mechanisms.

(+)-Macralstonidine **23** is composed of a macroline **5** and a *N*_a_-methyl sarpagine **124** unit. (+)-*N*_a_-Methyl sarpagine **124** (sarpagine unit) was isolated from *A. spectabilis* [31] and its total synthesis was accomplished by Zhao et al., as discussed earlier [83,84]. In a chemical sense, (+)-macralstonidine **23** should arise from the acid-mediated condensation of macroline **5** with (+)-*N*_a_-methyl sarpagine **124**. When macroline **5** was stirred with (+)-*N*_a_-methyl sarpagine **124** under mildly acidic conditions, the so formed cyclic ketal was identical to the authentic sample of macralstonidine **23** [31]. In this case too, the condensation followed by cyclic ketal formation can be explained by two different mechanisms. The straightforward Michael-type cyclization, which was discussed previously, is shown below (Scheme 10). On the other hand, the Friedel–Crafts alkylation-type mechanism that involved stabilization of the macroline enone with an oxonium anion is somewhat more probable from a chemical sense. As shown in Scheme 10, after initial intramolecular reaction of the C-17 hydroxyl function with the macroline ketone **5**, followed by acid-catalyzed dehydration, this process produced the oxonium species, which existed in two resonance stabilized forms (**71a** and **71b**). The electron-rich phenolic sarpagine **124** carbon atom could attack this electrophile from the C-9 position, which was *ortho* to the phenolic group. No alkylation at the C-11 position was observed. The so formed enol ether underwent acid-mediated rearrangement forming an oxonium intermediate **143,** which made it susceptible to nucleophilic attack by the phenolic oxygen atom furnishing the cyclic ketal present in macralstonidine **23**. Since macroline **5** and *N*_a_-methyl sarpagine **124** have both been synthesized in Milwaukee, this constitutes an enantiospecific total synthesis of (+)-macralstonidine **23** [76,79].

### 6.4. Biomimetic Total Synthesis of (+)-Macralstonine **24**

**Scheme 11:**
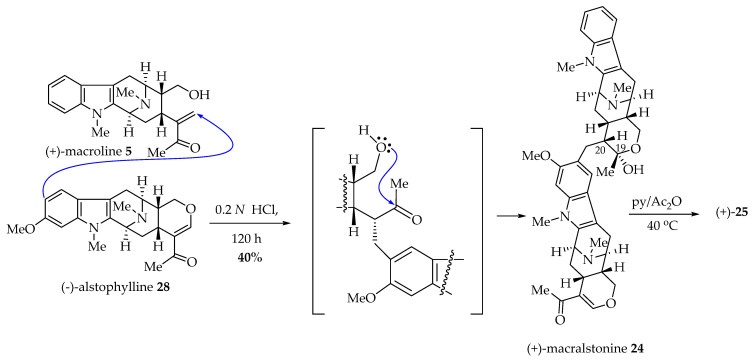
The biomimetic total synthesis of macralstonine **24**, and *O*-acetylmacralstonine **25**.

LeQuesne et al. [89] coupled natural (-)-alstophylline **28** and an excess of (+)-macroline **5** in aqueous 0.2*N* HCl solution at 20 °C for 120 h. The bisindole, (+)-macralstonine **24**, was obtained in 40% yield as a mixture of C-20 epimers, which later was converted into *O*-acetyl macralstonine **25** as the sole product after the treatment with acetic anhydride and pyridine [89]. LeQuesne et al. [89] confirmed the biosynthetic route (Scheme 11) of macralstonine **24** proposed by Schmid et al.; however the Friedel–Crafts alkylation process, as discussed above (Figure 5) is possible as well. Since the total synthesis of both macroline **5** and alstophylline **28** were accomplished by Liao et al., this constitutes the total synthesis of macralstonine **24**, and *O*-acetylmacaralstonine **25** by LeQuesne, Liao, Burke, and Cook et al. [76,89].

### 6.5. Partial Synthesis of (-)-Macrocarpamine **31**

**Scheme 12:**
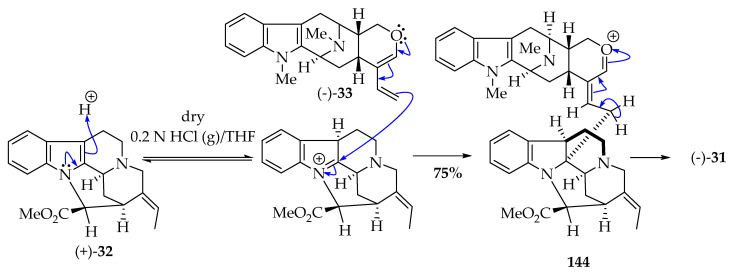
Partial synthesis of (-)-macrocarpamine **31**.

Gan et al. accomplished the partial synthesis of (-)-macrocarpamine **31** in stereospecific and enantiospecific fashion via the coupling reaction between the protonated natural (+)-pleiocarpamine **32** and synthetic (-)-anhydromacrosalhine-methine **33** (Scheme 12) [44]. The portion-wise addition of six equivalents of (-)-anhydromacrosalhine-methine **33** to (+)-pleiocarpamine **32** in 0.2 *N* dry HCl/THF, followed by basic workup (10% NH_4_OH), furnished (-)-macrocarpamine **31** in 75% yield [65]. It is important to stir pleiocarpamine **32** in acid to form the iminium ion first and then add (-)-**33** to it. Initial treatment of (-)-anhydromacrosalhine-methine **33** and (+)-pleiocarpamine **32** in aqueous and mild acidic conditions (0.2 *N* HCl) converted enol **33** into its hydrated product, inhibiting the desired product **144** formation. This was reported to be the first coupling reaction between an iminium ion and vinylogous enol ether of such type (**33**) and the first proton-mediated coupling reaction to obtain a bisindole via the coupling of two monomeric units from the *Alstonia* genus [65]. The total synthesis of (-)-macrocarpamine **31** has not been reported yet. Since the total synthesis of both of its monomeric units are now successfully completed, the formal total synthesis of (-)-macrocarpamine **31** is completed [17,44]. 

### 6.6. Partial Synthesis of (+)-Villalstonine **43**

**Scheme 13:**
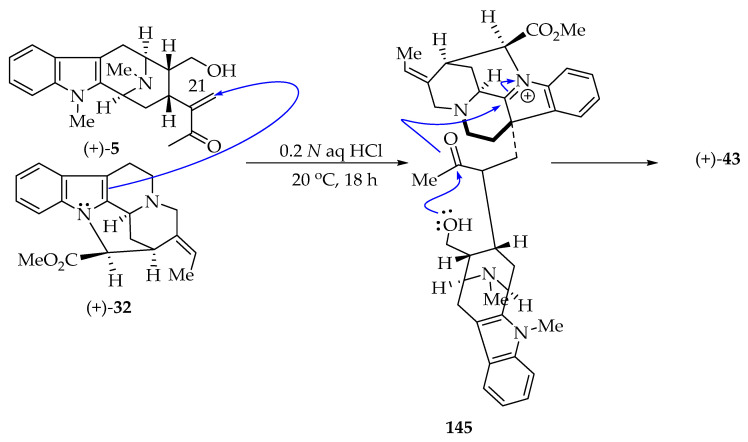
Biomimetic partial synthesis of (+)-villalstonine **43**.

**Scheme 14:**
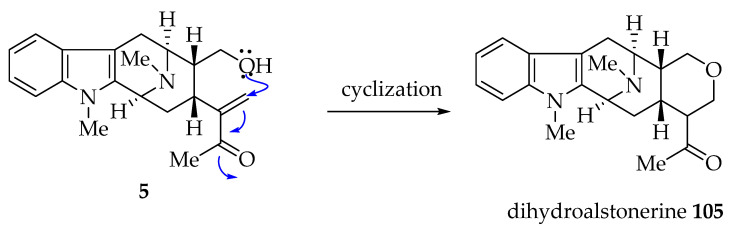
Intramolecular cyclization of macroline **5** into dihydroalstonerine **105**.

Schmid et al. described the degradation of (+)-villalstonine **43** into two monomeric indole alkaloids, (+)-pleiocarpamine **32** and the macroline base **5** [38]. Furthermore, Schmid anticipated forming a new acetal–amino acetal function in (+)-villalstonine **43** by electrophilic addition of the enone function of macroline **5** to the (+)-pleiocarpamine **32**, followed by ring closure. The formal total synthesis of (+)-villalstonine **43** has recently been accomplished, since Sato et al. [17] completed the total synthesis of *rac*-pleiocarpamine **32** and Liao, Bi, and Zhang prepared macroline **5** [45,90,91]. Initially, LeQuesne, Burke and Cook accomplished the partial synthesis of (+)-villalstonine **43** by a biomimetic-type coupling reaction, which was reported as the first among bisindole alkaloids from the *Alstonia* genus (Scheme 13 [51]. LeQuesne et al. treated (+)-pleiocarpamine **32** with (+)-macroline **5** in the presence of 0.2 *N* aqueous hydrochloric acid at 20 °C for 18 h to furnish (+)-villalstonine **43** as a single product on TLC [51]. The macroline **5** has a limited shelf life because it cyclizes intramolecularly to give the more stable dihydroalstonerine **105** (Scheme 14) [6].

The biomimetic synthesis of (+)-villalstonine **43** is thought to begin via electrophilic addition of the macroline enone **5** onto pleiocarpamine **32** from the *α*-face (less hindered) to give the intermediate **145** (Scheme 13) [51]. The intermediate **145** then forms a hemiacetal, after which the newly formed acetal sequentially attacks the iminium ion of the indoline from the less hindered side to furnish the acetal–amino acetal function in (+)-villalstonine **43** [51]. 

### 6.7. Improved Partial Synthesis of (+)-Villalstonine **43**

**Scheme 15:**
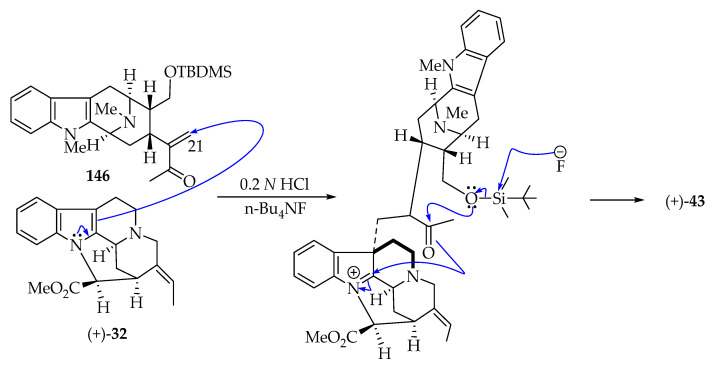
Improved partial synthesis of (+)-villalstonine **43**.

Because macroline **5** under acidic conditions forms part of the very stable dihydroalstonerine **105** (Scheme 14), the process was later modified by using the more stable macroline equivalent **146** [45]. The alcohol functional group in the macroline equivalent **5** was protected as its TBDMS ether **146** [79]. The natural (+)-pleiocarpamine **32** was received from Professor LeQuesne and then treated with the more stable macroline equivalent **146** in the presence of 0.2 *N* aqueous hydrochloric acid and fluoride ion (*tert*-butylammonium fluoride) for 24 h at 22 °C (Scheme 15). This procedure was followed by a basic workup with aqueous NH_4_OH to obtain (+)-villalstonine **43** as an oil in 60% condensation yield. Traces of (+)-pleiocarpamine **32** remained on TLC. The desilylation might occur before condensation to give macroline **146,** or the condensation may take place first as shown in Scheme 15 [6]. The spectroscopic data, including IR and NMR, TLC, and mass spectrometry of synthetic (+)-villalstonine **43,** were identical when compared to the authentic sample separately isolated from *A. muelleriana* by LeQuesne and Schmid et al. [38,51]. Since the total synthesis of both of its monomeric units are now successfully completed, the formal total synthesis of (+)-villalstonine **43** is completed [47,78]. 

## 7. C-19 Methyl-Substituted Sarpagine/Macroline/Ajmaline Alkaloids

**Figure 8:**
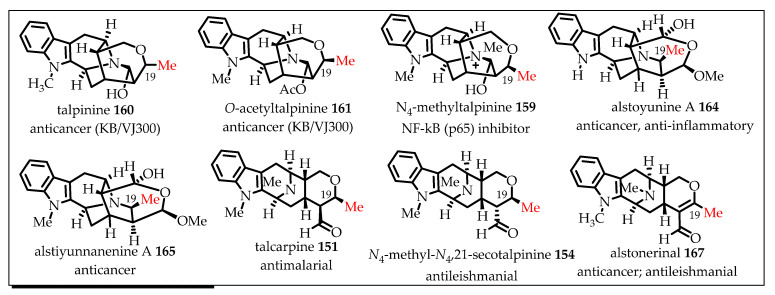
Representative examples of C-19 methyl-substituted monomeric sarpagine/macroline-related alkaloids from *Alstonia* species and their bioactivity.

The C-19 methyl-substituted sarpagine/macroline/ajmaline-type alkaloids are an emerging group of structurally and biosynthetically related alkaloids. To date, more than 70 monomeric indole alkaloids that belong to this sub-family have been isolated. In addition to the monomeric bases, several bisindole alkaloids consisting of at least one monomeric unit with a C-19 methyl group as their obligate constituent have been found in various medicinal plants worldwide (vide infra, Table 2). Bioactive C-19 methyl-substituted sarpagine/macroline bases and related alkaloids occur in various *Alstonia* species. The reported bioactivity of representative examples is listed herein (vide infra, Figure 8). It can be seen in Figure 8 that these alkaloids contain a stereogenic methyl function at the C-19 position and presumably originate from common or similar biosynthetic precursors. As a result, some common intermediates or core architecture should enable the total synthesis of these alkaloids. As illustrated earlier in Figure 7, macroline, sarpagine, and ajmaline alkaloids are essentially biosynthetic relatives, and they contain the azabicyclo[3.3.1]nonane core **93** present in their structures. Thus, any effective and divergent synthetic strategy should involve robust access to the common intermediates, preferably containing the azabicyclo[3.3.1]nonane core system **93**. The total synthesis of bisindoles containing the C-19 methyl function has not previously been reported. On the other hand, there has been notable progress in developing synthetic strategies for practical access to this important class of bioactive indole alkaloids [7,62].

### Enantiospecific Total Synthesis of C-19 Methyl-Substituted Sarpagine/Macroline Alkaloids via the General Strategy Developed in Milwaukee

**Scheme 16:**
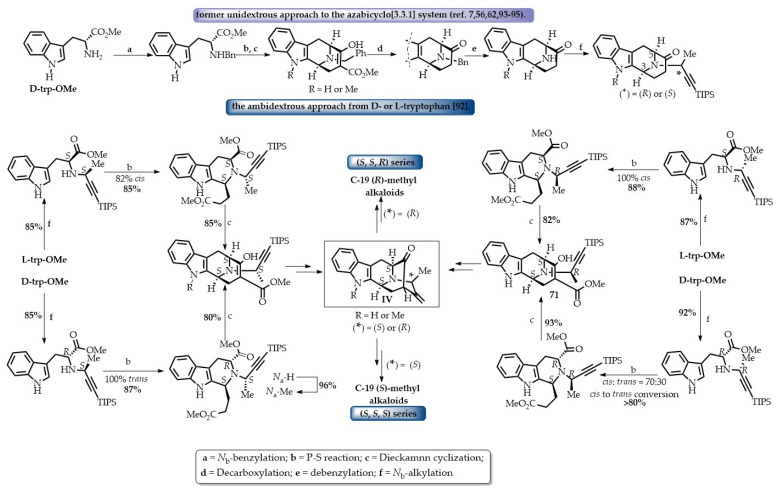
Improved general strategy toward the tetracyclic ketone intermediates of C-19 methyl-substituted alkaloids.

**Scheme 17:**
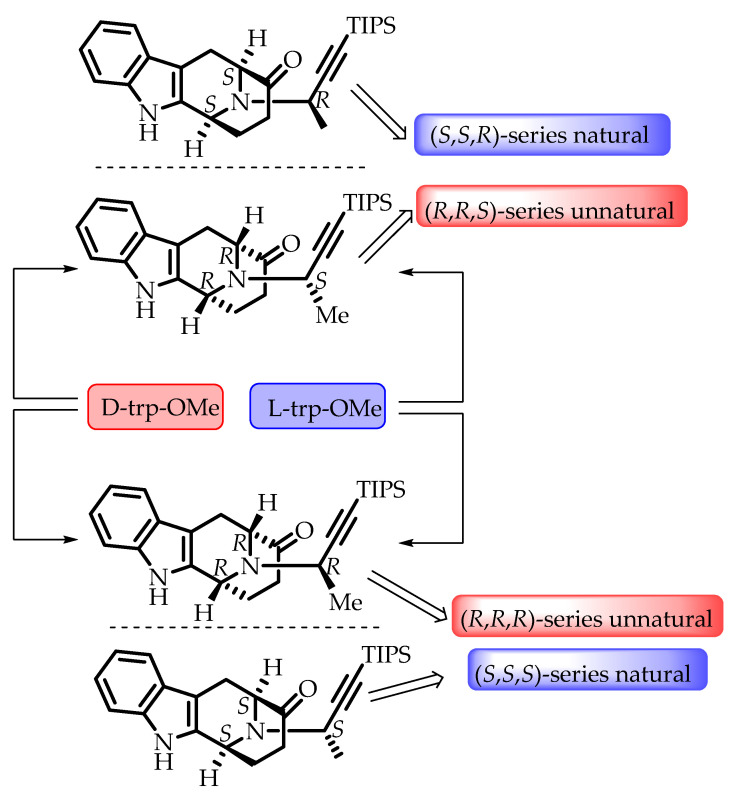
Access to both natural and unnatural enantiomers of C-19 methyl-substituted sarpagine/macroline indole alkaloids from the same chiral tryptophan.

**Figure 9:**
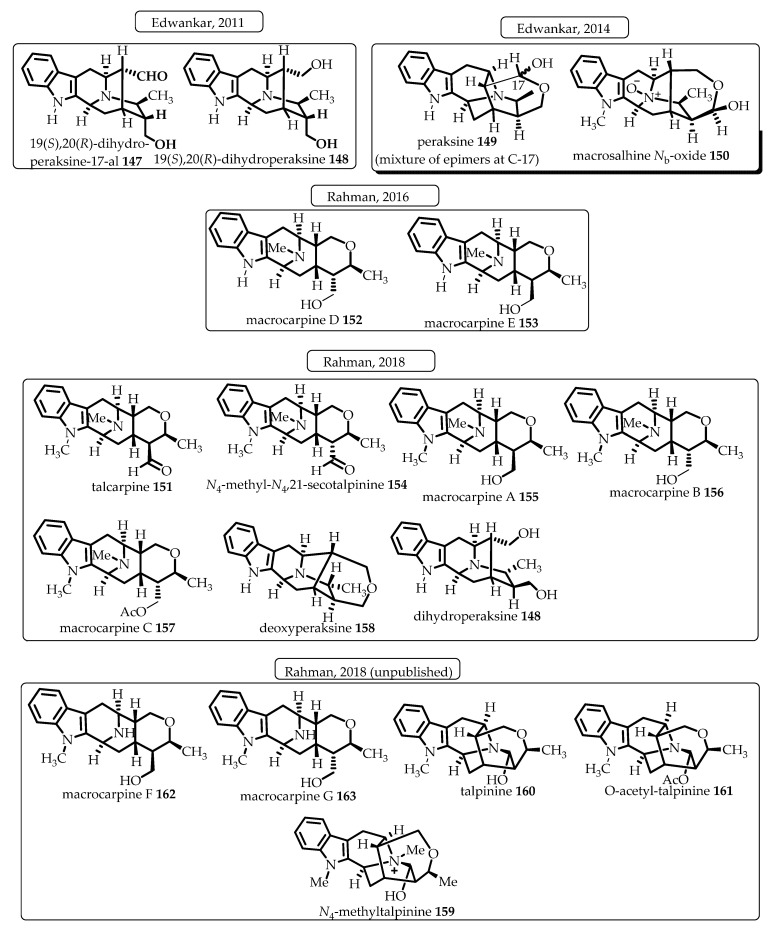
Examples of C-19 methylated alkaloids synthesized employing the general strategy developed herein.

During the development of an improved and shorter access to the tetracyclic ketone containing the azabicyclo[3.3.1]nonane core architecture, Rahman et al. developed an ambidextrous Pictet–Spengler (P-S)/Dieckmann protocol that provided efficient stereospecific access to the desired common intermediates towards the C-19 methyl-substituted sarpagine/macroline-type monomeric indole alkaloids [92]. The key tetracyclic intermediates for access to both the C-19 (*S*) or (*R*)-methyl-substituted alkaloids can now be synthesized either from D- or from L-tryptophan, at will, albeit at improved yield and in a route two steps shorter than the former strategy (Scheme 16). Further studies in this vein resulted in an illustration of the applicability of the so developed ambidextrous P-S strategy by accessing both enantiomers (individually) of the azabicyclo[3.3.1]nonane core structures, while starting from the same tryptophan auxiliary. One of the important implications of this ambidextrous P-S/Dieckmann protocol is that not only are the natural alkaloids containing both (*S*) and (*R*) C-19 methyl-containing alkaloids available but their enantiomers (unnatural alkaloids) can also be accessed using this strategy from the same chiral auxiliary (Scheme 17). To date, this unified general strategy has been illustrated in the total synthesis of more than a dozen alkaloids from this sub-family (Figure 9) [7,56,62,93,94,95]. A detailed discussion of the reported total synthesis of the individual monomeric alkaloids from this sub-family is out of the scope of this review. The enantiospecific total synthesis of the first example of sarpagine-type alkaloids isolated from the root-culture of *R. serpentina* that belong to this group was reported by Edwakar et al. [94]. These two monoterpenoid sarpagine alkaloids, 19(*S*),20(*R*)-dihydroperaksine-17-al **147** and 19(*S*),20(*R*)-dihydroperaksine **148** (Figure 9) represented a novel subgroup of sarpagine alkaloids, identified by Stöckigt et al. [96]. Afterwards, several other alkaloids including peraksine **149**, macrosalhine-N_b_-oxide **150**, and talcarpine **151** (formal synthesis) from this group were reported by employing the general strategy developed in Milwaukee [93]. Later, as a result of continued studies in this vein, Rahman et al. employed an efficient copper-mediated enolate-driven cross-coupling process that replaced the previous palladium-catalyzed coupling to gain an improved access to the core architecture of this subgroup of alkaloids and illustrated the process in the total synthesis of two monomeric macroline alkaloids, macrocarpines D **152** and E **153** [62]. In 2018, Rahman et al. reported an improved strategy for a two-step shorter and higher overall yield to achieve access to the key tetracyclic ketone intermediates. The total synthesis of a number of sarpagine and macroline alkaloids, including talcarpine **151**, *N*_4_-methyl-*N*_4_,21-secotalpinine **154**, macrocarpines A–C (**155–157**), dihydroperaksine **148**, and deoxyperaksine **158,** was completed and reported in the same paper. This also corrected the reported optical rotations of *N*_4_-methyl-*N*_4_,21-secotalpinine **154** and macrocarpine A **155** [7]. Afterwards, the focus turned toward the development of the ambidextrous Pictet–Spengler approach for accessing natural alkaloids either from D- or L-tryptophan, at will. Besides, both natural and unnatural enantiomers would be accessible from the same chiral starting material. Several more alkaloids from this subgroup, including the potent NF-kB inhibitor N_4_-methyltalpinine **159**, anticancer alkaloids talpinine **160** and O-acetyl talpinine **161**, as well as the macrocarpines F-G (**162–163**), have been synthesized and will be reported in due course (Figure 9) [97].

Any member of this group would be accessible employing this general strategy, albeit most of these alkaloids await their total synthesis. In addition, no bisindole alkaloid from this group has been synthesized, to date, but the building blocks are now in place. The important bioactivity of a number of alkaloids from this group or their unnatural enantiomers (both monomeric and bisindole) by combining different bioactive monomeric units might result in novel chemical entities, which are unavailable in normal biosynthetic processes. In this respect, the enantiomers of the bioactive monomeric C-19 methylated alkaloids would also be available from either of the tryptophan esters at will. Enantiomers may have similar or even better properties depending on their metabolism, and this holds true for bisindoles, as well as their mismatched pairs.

## 8. Unnatural (Enantiomers or Synthetic Derivatives) Alkaloids in Drug Discovery

Natural molecules or compounds are usually chiral molecules that are the secondary metabolites used in plants or organisms, perhaps as defense mechanisms. The plants or organisms have evolved to produce only particular enantiomers, which results in the isolation and screening of only particular enantiomers, i.e., the natural enantiomers. Optically pure drugs from natural sources exhibit desired biological activity usually in particular enantiomeric forms, for example, morphine and reserpine [98]. Anticipated pharmacological effects can be achieved by applying absolute stereochemistry or enantioselectivity of compounds in many enzymatic reactions [98]. Logically, the unnatural enantiomers might be as good as the natural enantiomers or even better in activity and toxicity profile, depending on the metabolism. For example, a drug in WHO’s essential list, lamivudine, an unnatural deoxycytidine analog, has saved millions of lives after its approval by the FDA in 1995 [99]. The unnatural L-nucleoside-based drug, emtricitabine, exhibited 100 times greater potency and longer duration of action than its natural D-nucleoside analog [92,99,100]. Among 185 novel small molecules approved by the FDA since 1981 for treatment of cancer, 120 (64.9%) are from a natural source, natural product mimetic, or natural product pharmacophore-inspired synthetic drug [1]. Notably, several C’-20 urea derivates of the robust clinical drug vinblastine are reported to be 10–200 times more active than the natural vinblastine analogs themselves against various human tumor cell lines [101]. When compared to the original compound, unnatural/synthetic drug candidates such as analogs of bryostatin, vincristine, and vinblastine might exhibit excellent biological activity [102,103,104]. The modified vinca alkaloids (fluorine substitution at C10′) 10′-fluorovinblastine and 10′-fluorovincristine exhibited substantially stronger activity than the natural microtubule inhibitors vinblastine and vincristine against the sensitive and vinblastine-resistant tumor cell lines, in the study from the Boger group [105]. Thus, it is crucial to discover the pharmacological effects of derivatives and unnatural enantiomers of natural products.

## 9. Conclusions

Natural products and nature-inspired molecules are expected to continue playing an indispensable role in modern drug discovery. Compared to their monomeric units, bisindoles are more potent in many cases. Bisindole alkaloids including semisynthetic derivatives have been found to possess significant bioactivity, including anticancer, antileishmanial, and antimalarial properties, and thus are promising leads for nature-inspired drug discovery and development. Unnatural medicinal compounds formed by combining bioactive mismatched monomeric units can furnish novel medicinal compounds. Incorporating unnatural enantiomers of monomeric alkaloids into bisindoles can provide access to novel and unnatural bioactive compounds that may have better activity and in vivo stability depending on their metabolism.

## Figures and Tables

**Table 1:** Indole alkaloids (monomeric units) in bisindoles from *Alstonia* species that are reviewed herein.

Types	Indole Alkaloids	
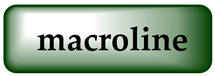	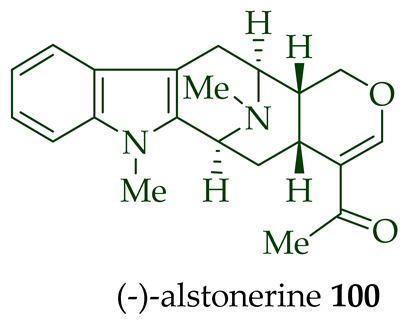	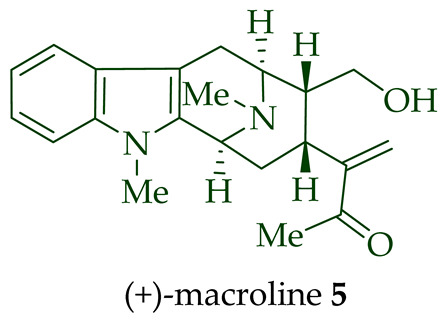
	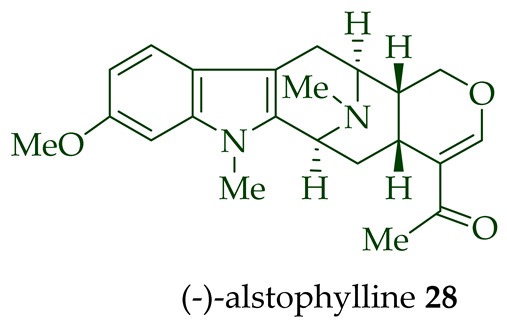	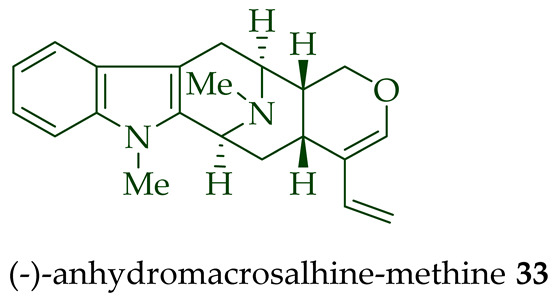
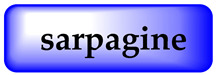	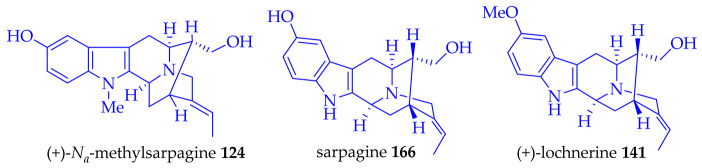	
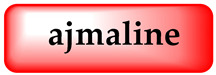	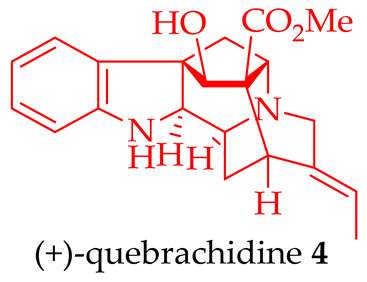	
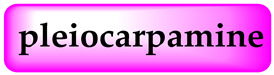	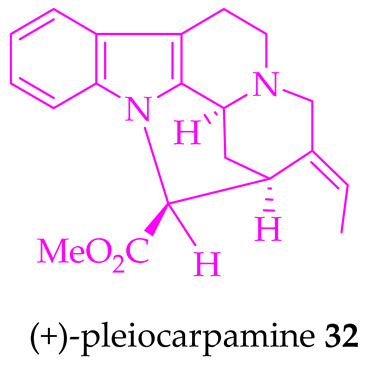	

**Table 2:** Bisindoles from *Alstonia* species that are reviewed herein.

Bisindole Alkaloids Reviewed Herein		
Macroline-macroline		
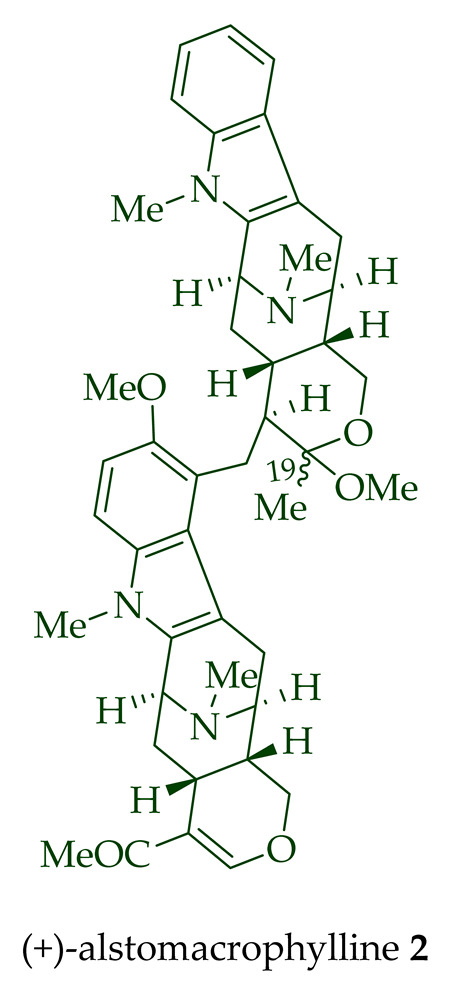	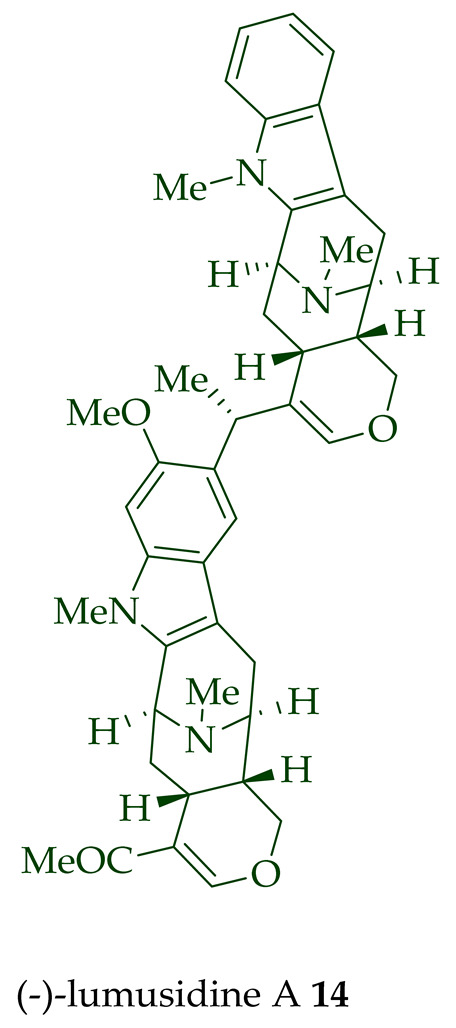	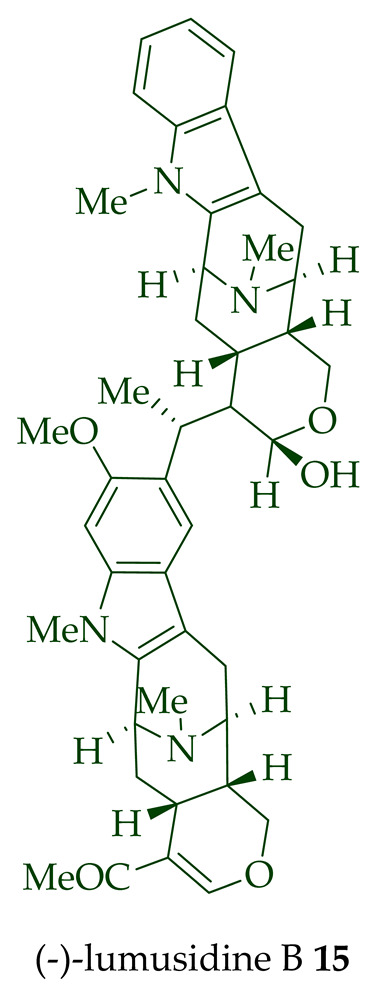
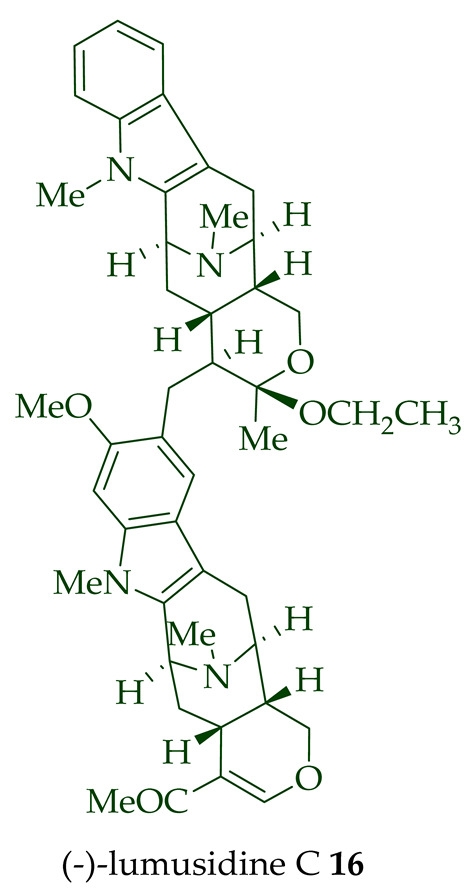	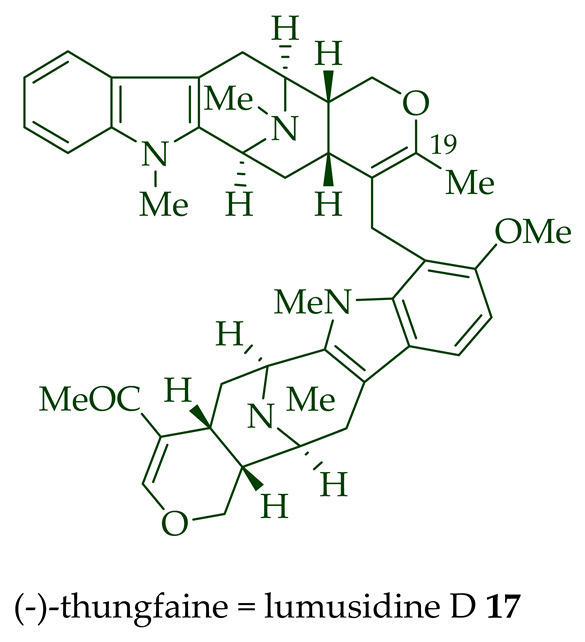	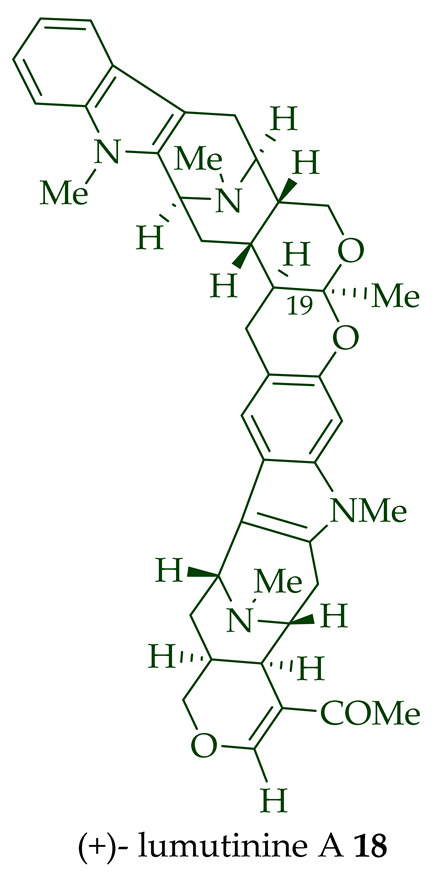
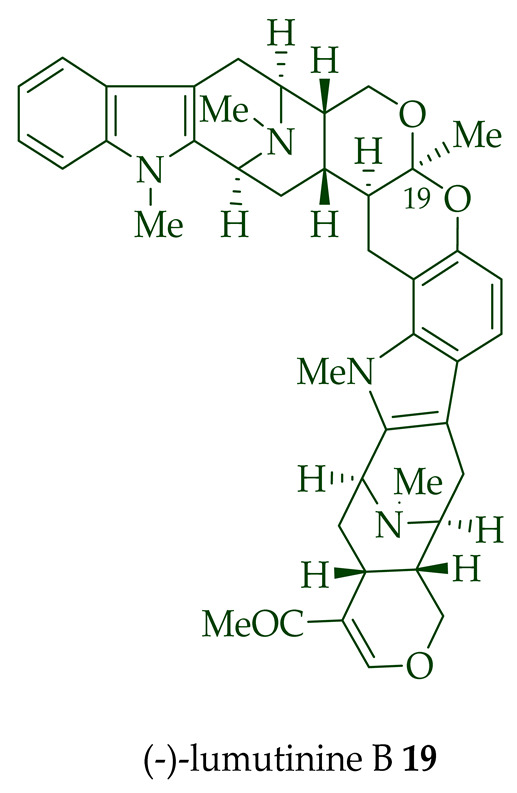	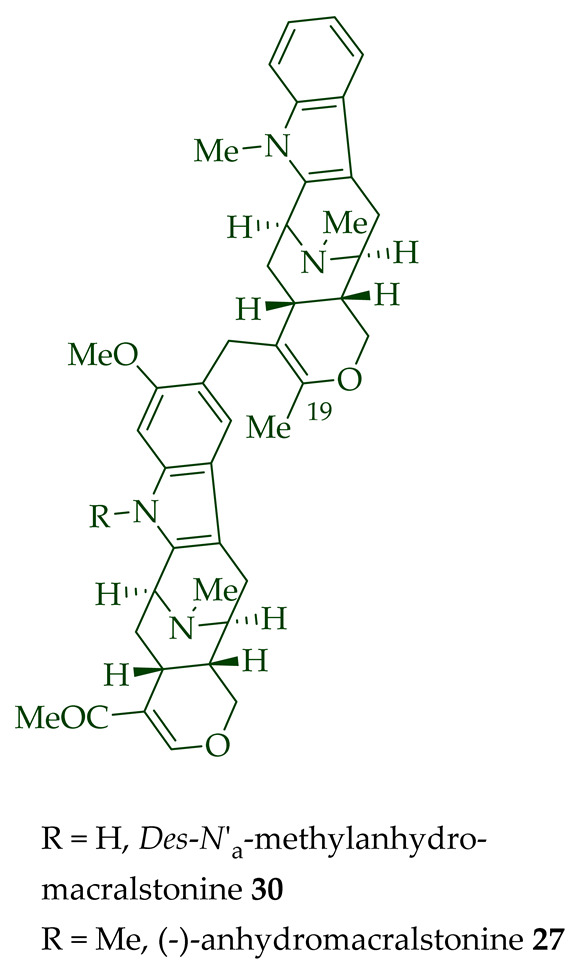	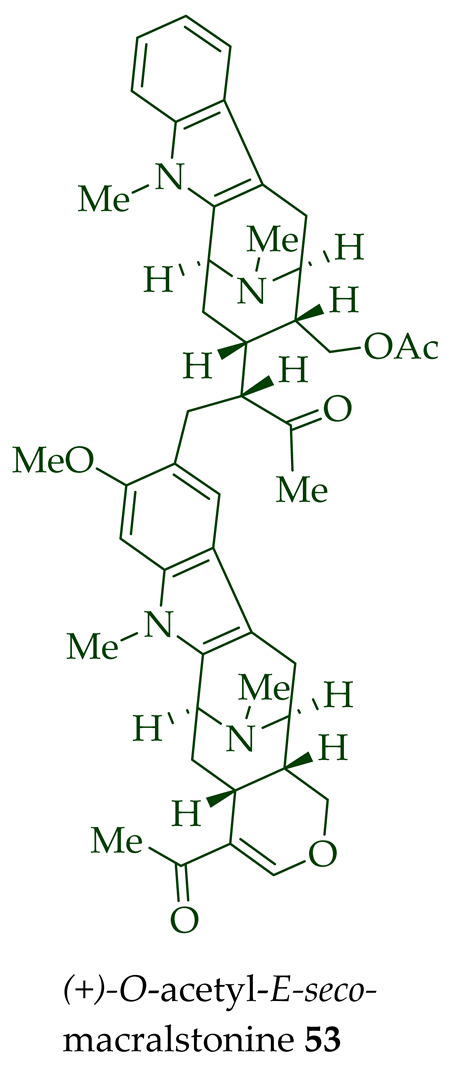
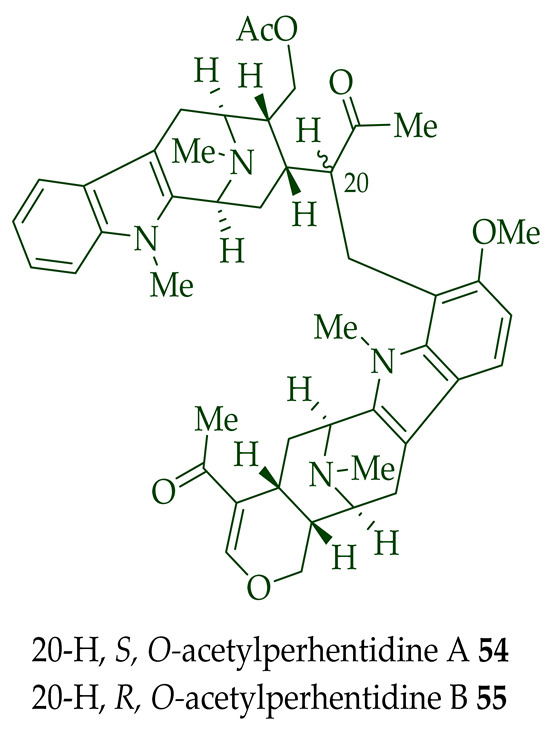	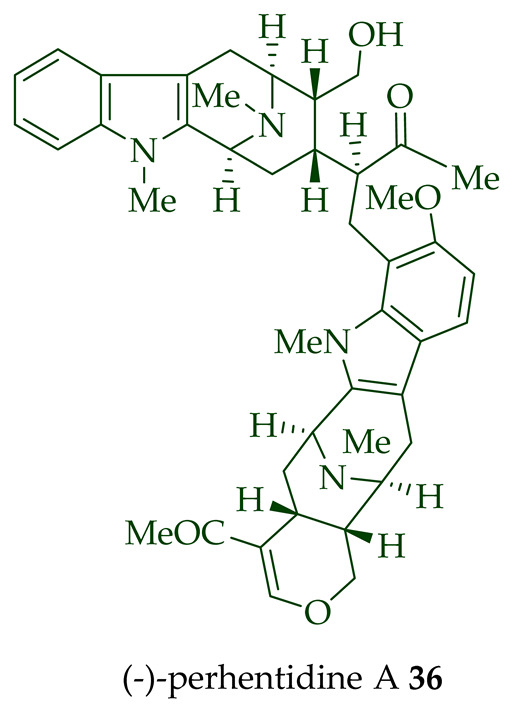	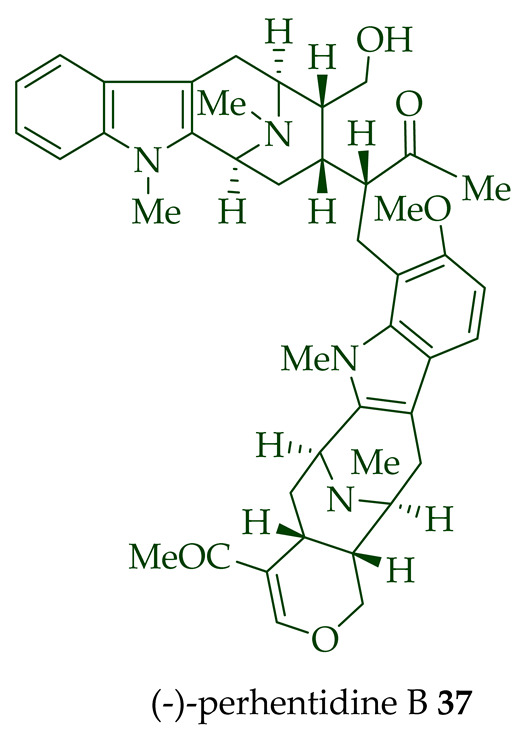
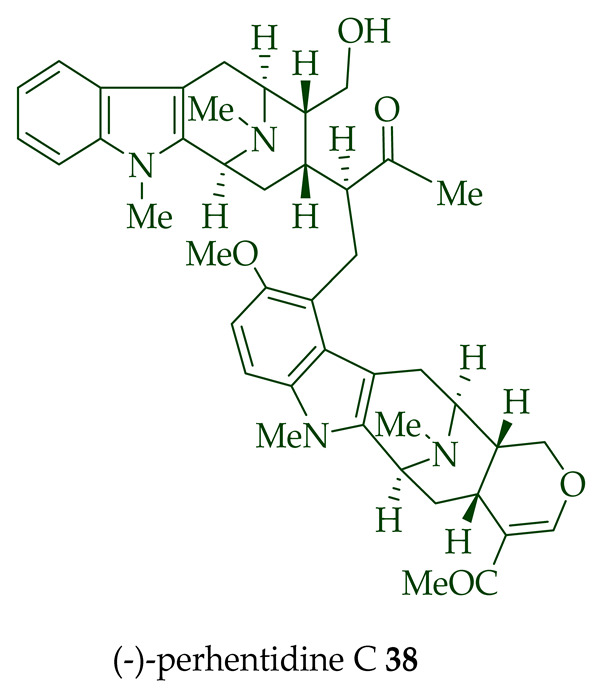	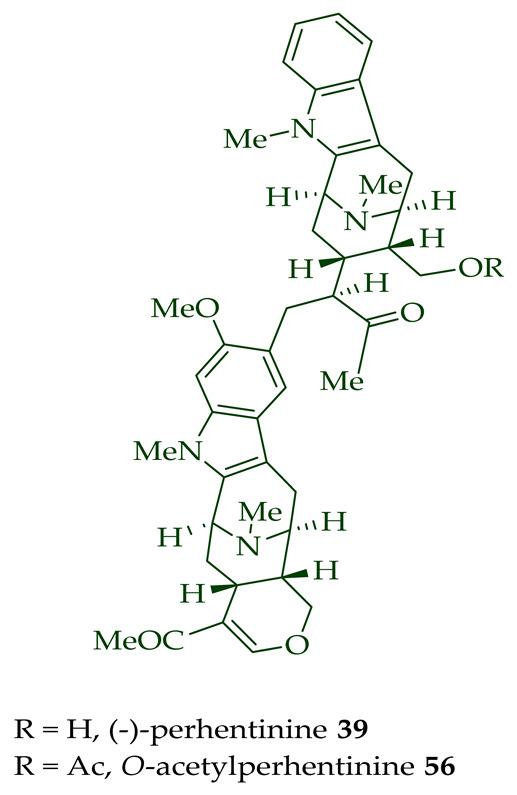	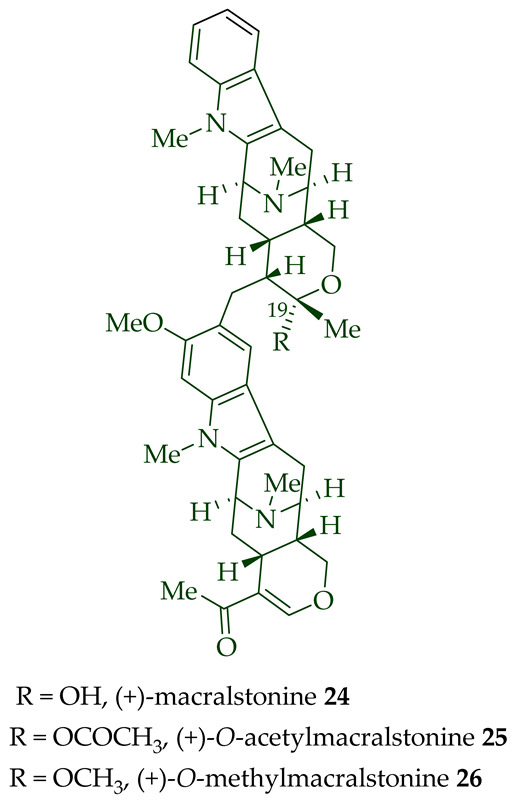
Macroline-sarpagine		
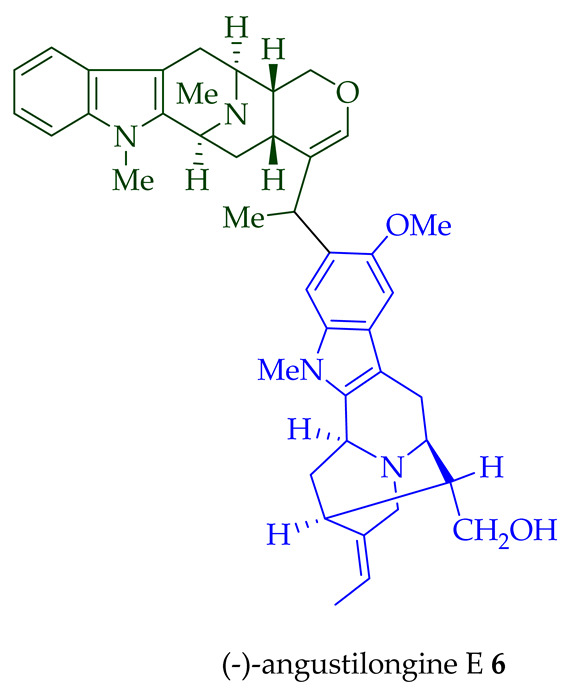	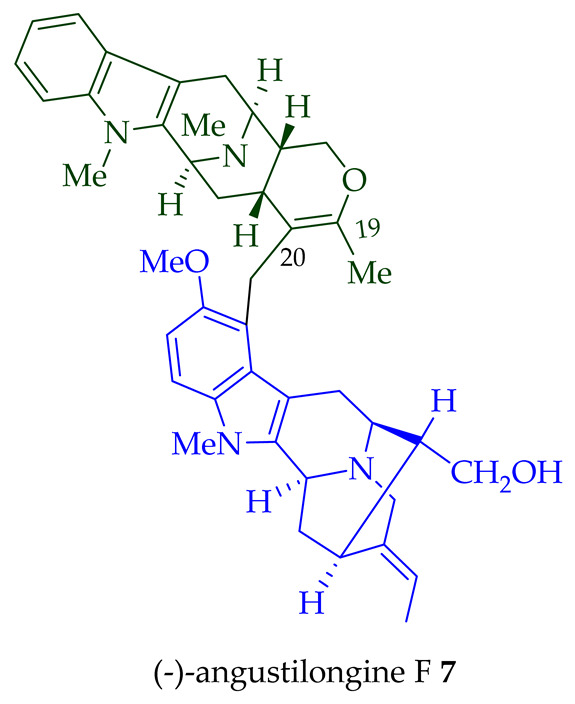	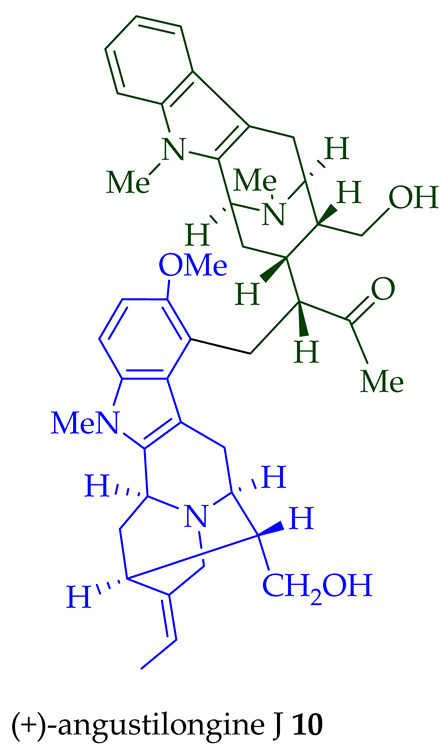
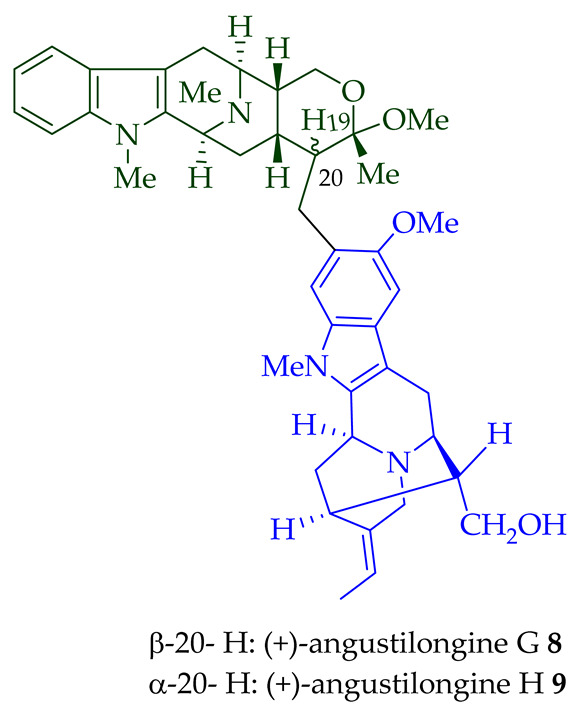	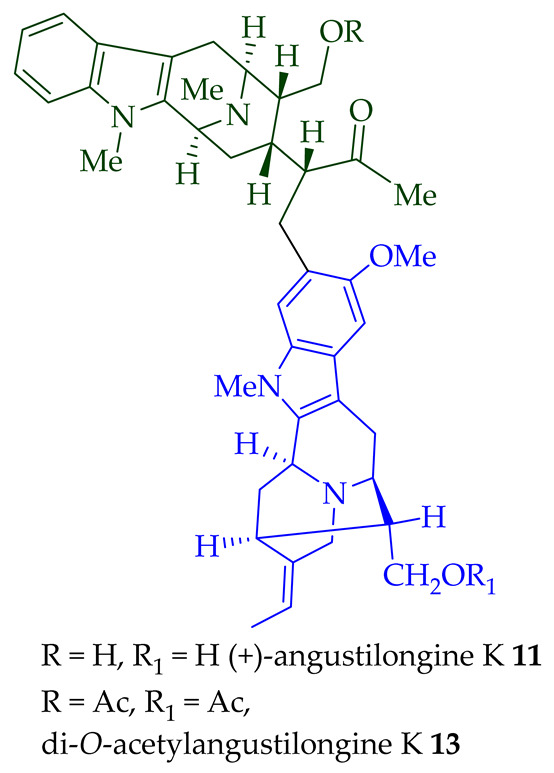	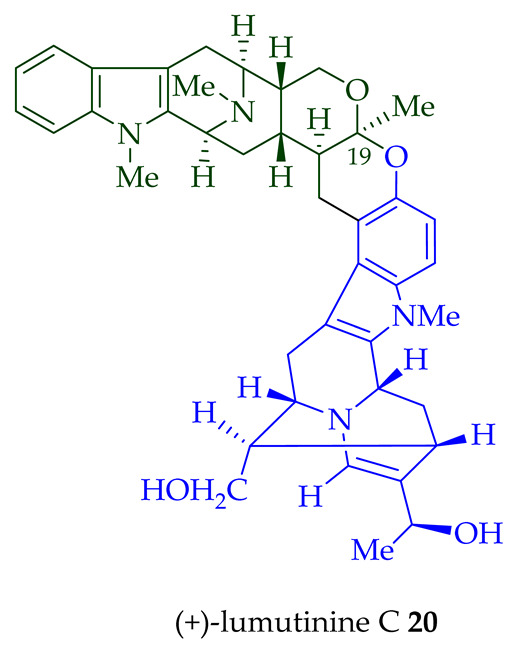
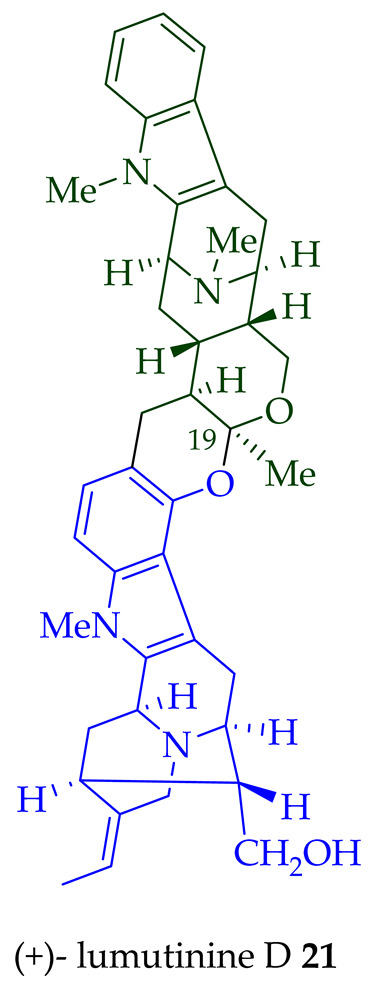	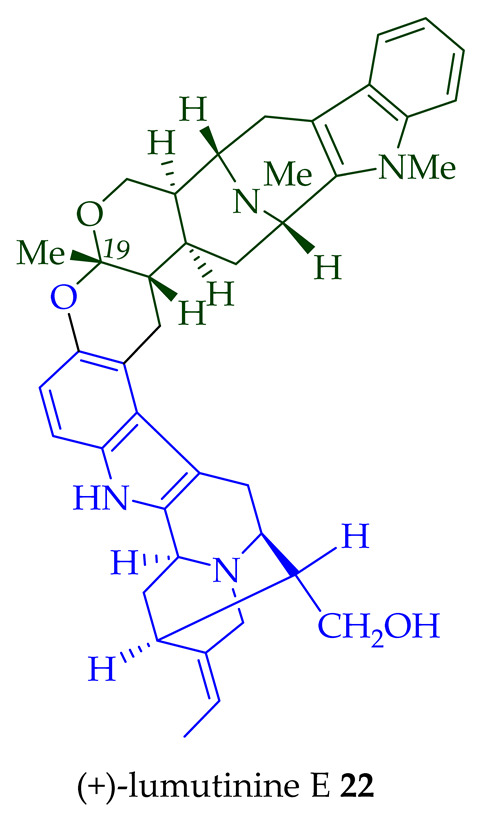	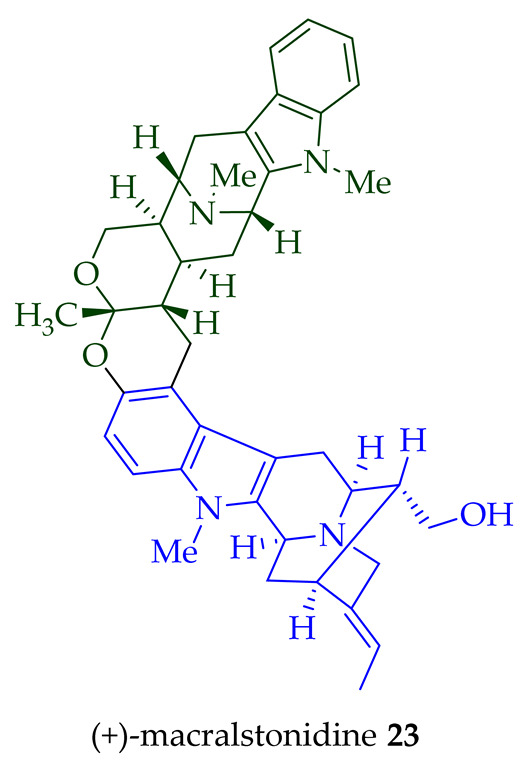
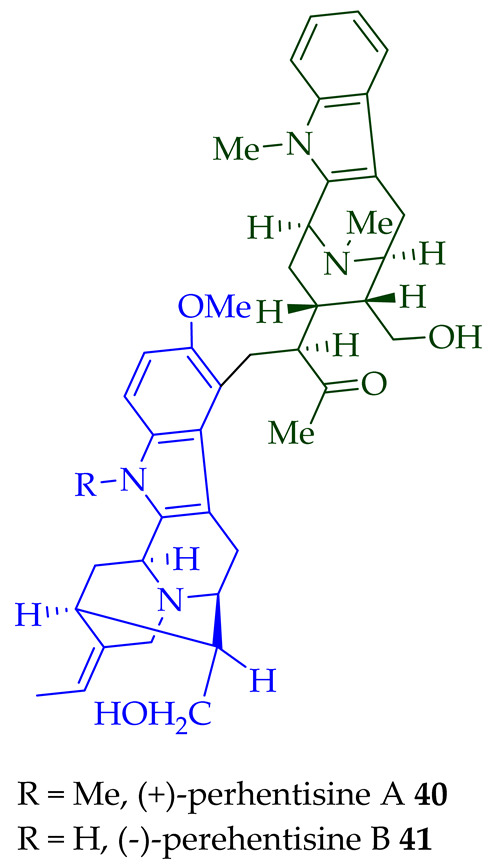	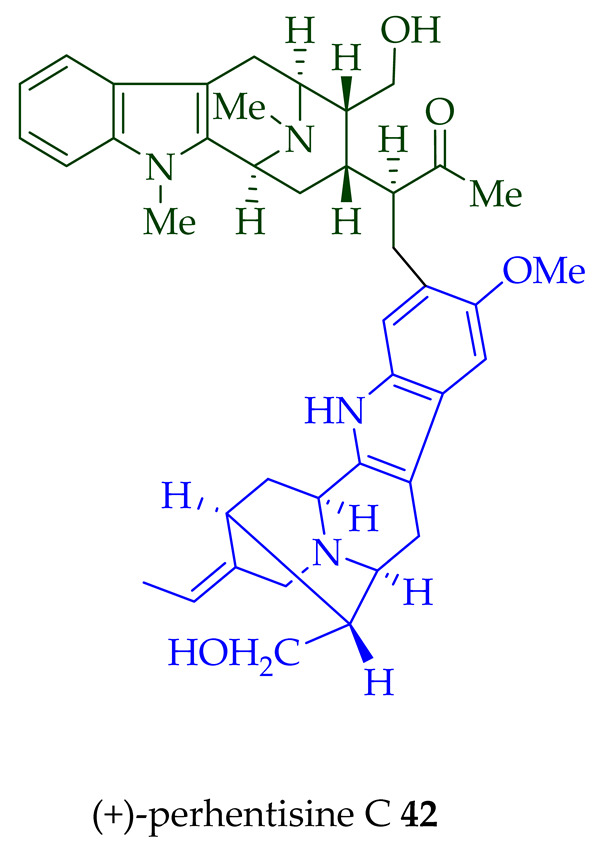	
Macroline-ajmaline		
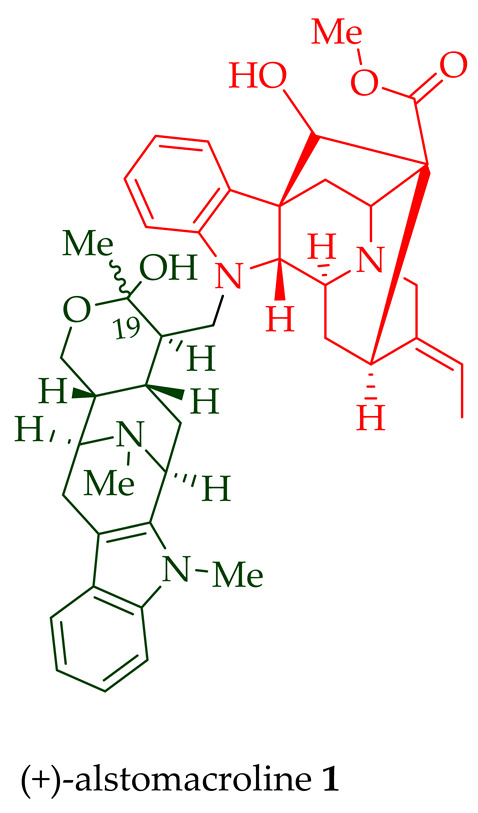	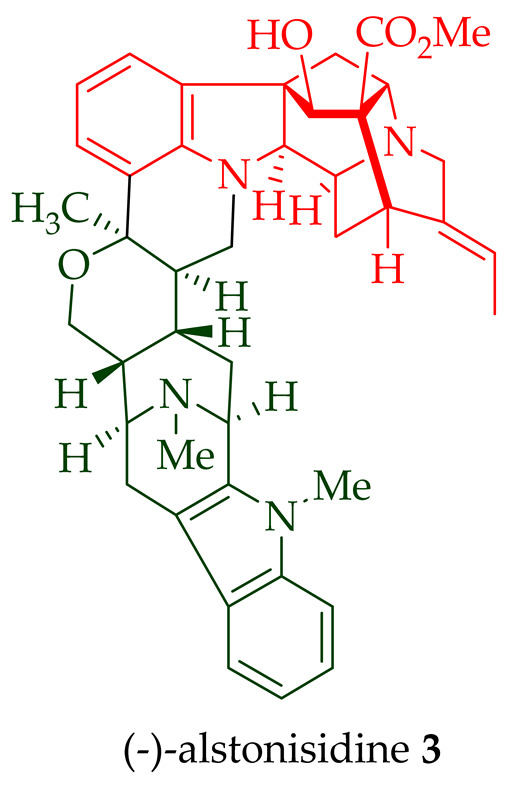	
Macroline-pleiocarpamine		
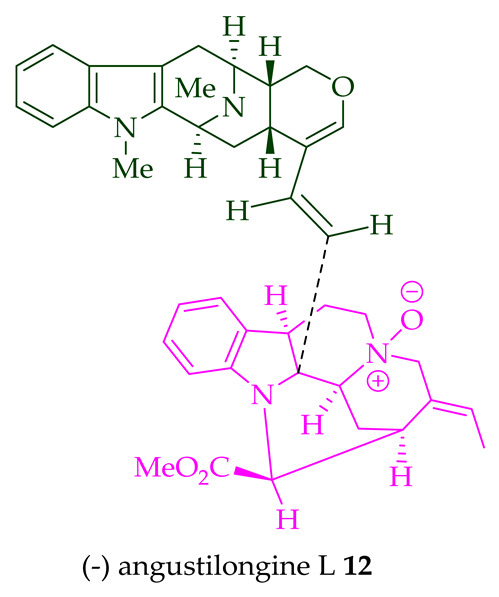	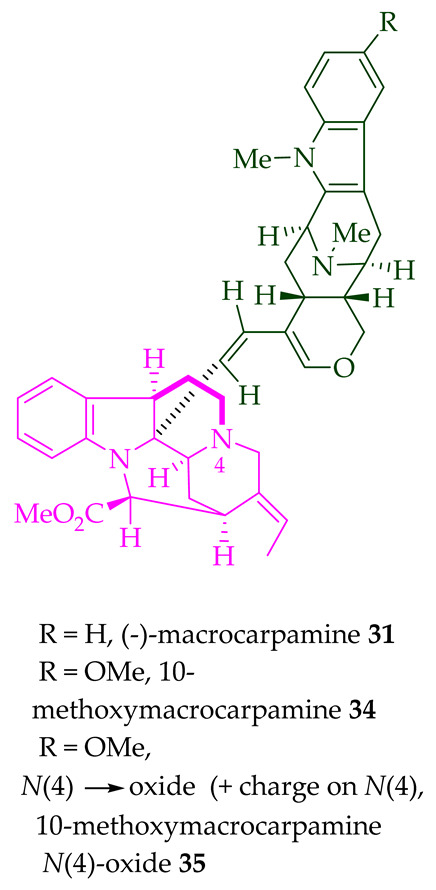	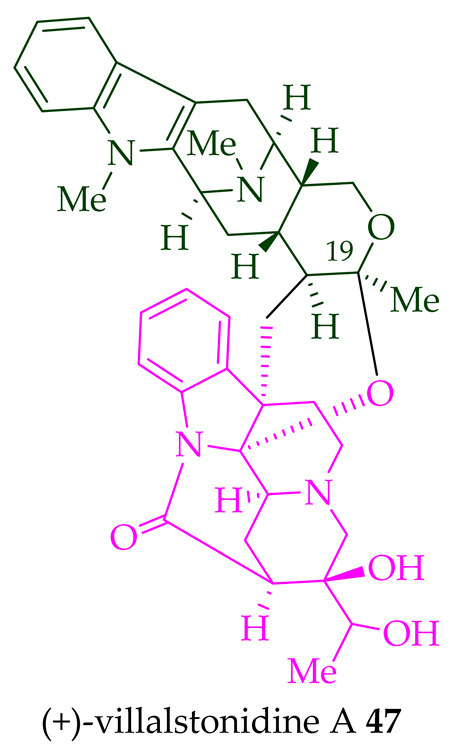
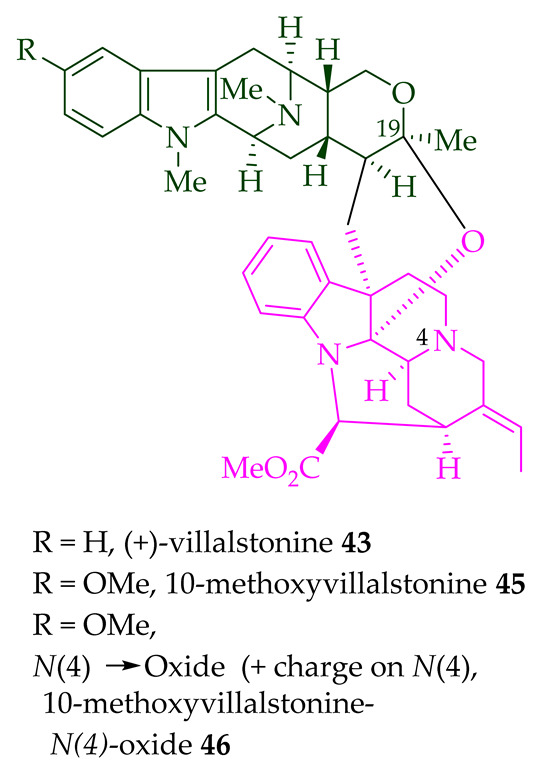	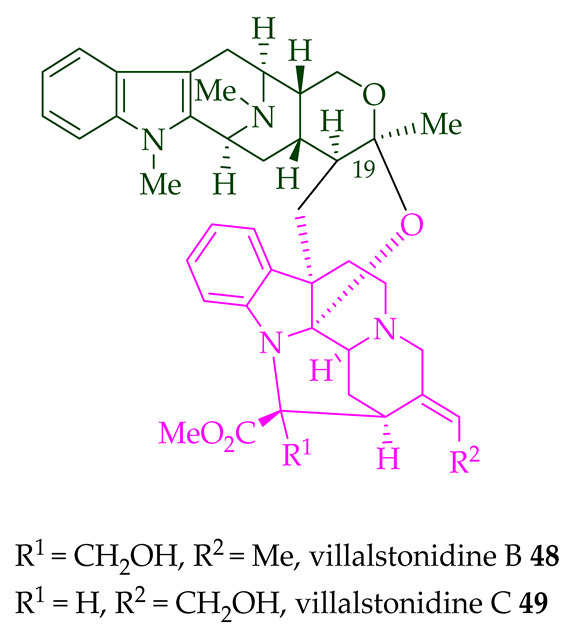	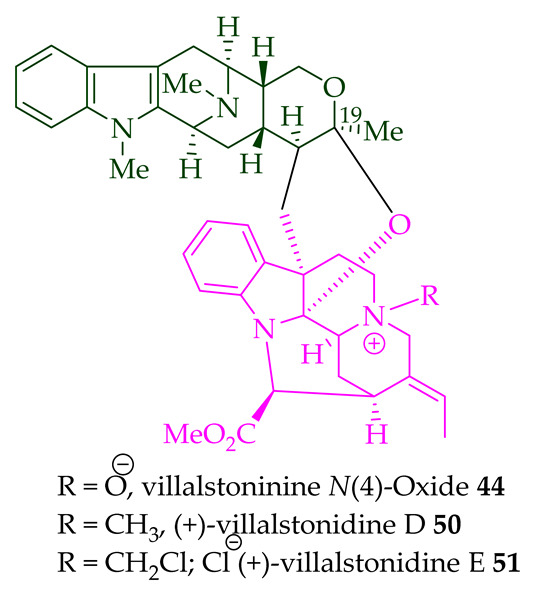
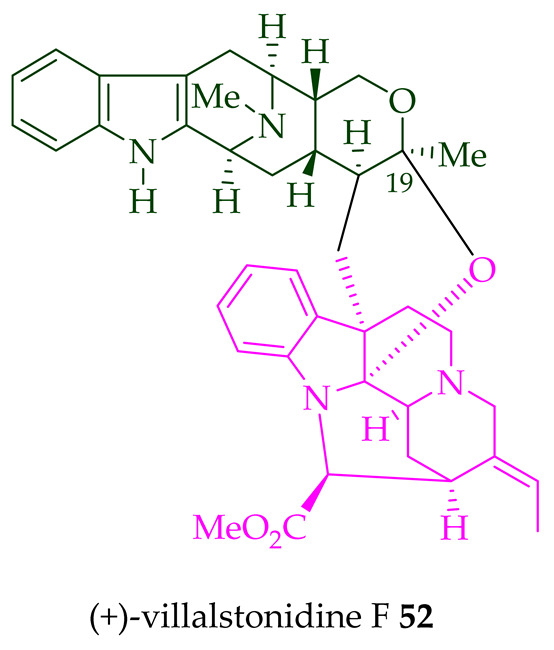		

**Table 4:** Bioactivity of bisindole alkaloids (including semisynthetic derivatives) from *Alstonia* species.

Bisindoles	Bioactivity	References
(+)-Alstomacroline **1**	Antimalarial, with IC_50_ values of 1.12 ± 0.35 and 10.0 ± 0.4 μM against the K1 strain and T9-96 strain of *P. falciparum*, respectively.	[20]
(+)-Alstomacrophylline **2**	Antimalarial, with an IC_50_ value of 1.10 ± 0.30 μM against the K1 strain of *P. falciparum*.	[20]
Angustilongines E, F, G, H, J, and K (**6**–**11**)	Anticancer, cytotoxic against various human cancer cell lines including KB, vincristine-resistant KB, HCCT 116, PC-3, MDA-MB-231, LNCaP, MCF7, HT-29, and A549 cells with IC_50_ values ranging from 0.02 to 9.0 μM.	[5]
(−)-Lumusidine A, B, and C (**14**–**16**)	Anticancer, moderately cytotoxic in KB/VJ300 cells with IC_50_ values of 0.16, 0.70, and 1.19 μg/mL (μM), respectively. The assay with 0.12 μM added vincristine did not influence KB/VJ300 cell growth.	[18]
(−)-Lumusidine D **17**	Anticancer, cytotoxic in KB/VJ300 cells with an IC_50_ value of 5.03 μg/mL (μM). The assay with 0.12 μM added vincristine did not influence KB/VJ300 cell growth.	[18]
Lumutinine A, B, C, D, and E (**18**–**22**)	Anticancer, moderately cytotoxic in KB/VJ300 cells with IC_50_ 0.21, 0.10, 4.61, 3.93, and 2.74 μg/mL (μM) values, respectively. The assay with 0.12 μM added vincristine did not influence KB/VJ300 cell growth.	[18]
(+)-Macralstonine **24**	Anticancer, strongly cytotoxic in KB/VJ300 cells with an IC_50_ 1.71 μg/mL (μM) value. The assay with 0.12 μM added vincristine did not influence KB/VJ300 cell growth.	[18]
	Antimalarial, active against the K1 strain of *P. falciparum* with an IC_50_ 8.92 ± 2.95 μM value.	[20]
(−)-Anhydromacralstonine **27**	Anticancer, moderately cytotoxic in KB/VJ300 cells with an IC_50_ value of 0.44 μg/mL (μM). The assay with 0.12 μM added vincristine did not influence KB/VJ300 cell growth.	[18]
(+)-*O*-Acetyl macralstonine **25**	Antimalarial, with IC_50_ values 0.53 ± 0.09 and 12.4 ± 1.6 (μM) against the K1 strain and T9-96 strain of *P. falciparum*, respectively.	[20]
(+)-*O*-Methyl macralstonine **26**	Antimalarial, active against the K1 strain of *P. falciparum* with an IC_50_ 0.85 ± 0.20 μM value.	[20]
*O*-Acetyl-*E-seco*-macralstonine **53**	Anticancer, strongly cytotoxic in KB/VJ300 cells with an IC_50_ value of 0.27 μg/mL (μM). The assay with 0.12 μM added vincristine did not influence KB/VJ300 cell growth.	[18]
(−)-Perhentidine A **36** and (-)-perhentidine B **37**	Anticancer, strongly cytotoxic in KB/VJ300 cells with IC_50_ values of 2.29 and 0.84 μg/mL (μM), respectively. The assay with 0.12 μM added vincristine did not influence KB/VJ300 cell growth.	[18]
(−)*-O*-Acetylperhentidine A **54** and (-)-*O*-Acetylperhentidine B **55**	Anticancer, strongly cytotoxic in KB/VJ300 cells with IC_50_ 0.36 and 0.28 μg/mL (μM) values, respectively. The assay with 0.12 μM added vincristine did not influence KB/VJ300 cell growth.	[18]
(−)-Perhentinine **39** and *O*-Acetylperhentinine **56**	Anticancer, cytotoxic in KB/VJ300 cells with IC_50_ values of 0.52 and 0.30 μg/mL (μM), respectively. The assay with 0.12 μM added vincristine did not influence KB/VJ300 cell growth.	[18]
(+)-Macralstonidine **23**	Anticancer, moderately cytotoxic in KB/VJ300 cells with an IC_50_ value of 0.13 μg/mL (μM). The assay with 0.12 μM added vincristine did not influence KB/VJ300 cell growth.	[18]
(+)-Macrocarpamine **21**	Anticancer, strongly cytotoxic in KB/VJ300 cells with an IC_50_ value of 0.53 μg/mL (μM). The assay with 0.12 μM added vincristine did not influence KB/VJ300 cell growth.	[18]
	Strong antimalarial activity against the K1 strain of *P. falciparum* with an IC_50_ value of 0.36 ± 0.06 μM. Active against the T9-96 strain of *P. falciparum* with an IC_50_ >39 μM value.	[20]
	Strong antiprotozoal activity in vitro against *E. histolytica* and *P. falciparum* with ED_50_ 8.12 (95% C.I.) μM and ED_50_ 9.36 (95% C.I.) μM values, respectively.	[43]
(+)-Villalstonine **43**	Anticancer, cytotoxic in KB/VJ300 cells with an IC_50_ value of 0.42 μg/mL (μM). The assay with 0.12 μM added vincristine did not influence KB/VJ300 cell growth.	[18]
	Anticancer, cytotoxic against the HT-29 cell line with an ED_50_ 8.0 μM value (paclitaxel was used as the positive control).	[39]
	Antimalarial, with IC_50_ values of 0.27 ± 0.06 and 0.94 ± 0.07 μM against the K1 strain and T9-96 strain of *P. falciparum*, respectively.	[20]
	Antiamoebic activity against *E. histolytica* with an ED_50_ of 2.04 μM.	[43]
Villalstonine *N*(4)-oxide **44**	Antileishmanial activity against promastigotes of *L. mexicana* with an IC_50_ value of 80.3 μM (amphotericin B was used as the positive control).	[39]
	Antimalarial, active against the K1 strain of *P. falciparum* with an IC_50_ 10.7 ± 1.9 (μM) value.	[20]
(+)-Villalstonidine B **48** and (+)-villalstonidine F **52**	Anticancer, strongly cytotoxic in KB/VJ300 cells with IC_50_ values of 0.35 and 5.64 μg/mL (μM), respectively. The assay with 0.12 μM added vincristine did not influence KB/VJ300 cell growth.	[18]
(+)-Villalstodinine D **50**	Antileishmanial, active against promastigotes of *L. mexicana* with an IC_50_ value of 120.4 μM (amphotericin B was used as the positive control).	[39]
(+)-Villalstonidine E **51**	Anticancer, cytotoxic against HT-29 cell lines with an ED_50_ 6.5 μM value (paclitaxel was used as the positive control).	[39]
	Antileishmanial against promastigotes of *L. mexicana* with an IC_50_ 78 μM value (amphotericin B was used as the positive control).	[39]

## Data Availability

Not applicable.
